# Synthesis and Host–Guest Properties of Acyclic Pillar[*n*]naphthalenes

**DOI:** 10.3389/fchem.2019.00828

**Published:** 2019-12-03

**Authors:** Yuanyin Jia, Ming Dong, Bin Wang, Chunju Li

**Affiliations:** ^1^School of Chemical and Environmental Engineering, Shanghai Institute of Technology, Shanghai, China; ^2^Key Laboratory of Inorganic-Organic Hybrid Functional Material Chemistry, Ministry of Education, Tianjin Key Laboratory of Structure and Performance for Functional Molecules, College of Chemistry, Tianjin Normal University, Tianjin, China; ^3^Department of Chemistry, Center for Supramolecular Chemistry and Catalysis, Shanghai University, Shanghai, China

**Keywords:** pillararenes, calixarenes, acyclic hosts, molecular recognition, host-guest chemistry

## Abstract

Here we report a new class of synthetic receptors, acyclic pillar[*n*]naphthalene (*n* = 2–4, **Dimer**, **Trimer**, and **Tetramer**) oligomers, which are made up of 2,3-diethoxynaphthalene units linked by methylene bridges at the 1- and 4-positions. They can be synthesized through a one-step condensation of 2,3-diethoxynaphthalene monomer and paraformaldehyde in the presence of BF_3_•(Et)_2_O catalyst. The crystal structure of **Tetramer** has an interesting pseudo-cycle shaped structure in the solid state. Their complexation behaviors toward several organic ammonium cations (**1**^+^-**15**^+^) and electron–deficient neutral guests (**16**–**17**), were examined by means of ^1^H NMR spectroscopy. **Tetramer** shows good host-guest properties toward the ammonium guests, giving association constants (*K*_a_) in the magnitude of 10^2^-10^4^ M^−1^, which are comparable with those for some macrocyclic hosts.

## Introduction

**Scheme 1:**
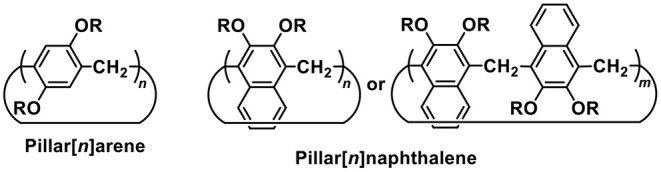
Structures of pillar[*n*]arenes and our designed pillar[*n*]naphthalenes.

Since the discover of crown ethers, the development of hosts for recognizing various guest species has mainly focused on macrocyclic structures (Cram, 1988; Lehn, 1988; Pedersen, 1988; Gong et al., 2010; Chun et al., 2013; Jurícek et al., 2014; Liu et al., 2019). Methylene–bridged macrocyclic arenes, for example calixarenes (Baldini et al., 2007; Guo and Liu, 2012), pillararenes (Ogoshi et al., 2008; Xue et al., 2012; Wang et al., 2016; Yang et al., 2016), coronarenes (Wang, 2018), helic[6]arene (Zhang et al., 2016), biphenarenes (Chen et al., 2015; Dai et al., 2017; Li et al., 2019; Wang et al., 2019b), and etc. (Guo et al., 2018; Luo et al., 2018; Ma et al., 2018) have been widely used in host-guest chemistry, self-assembly materials, and biomedical field (Song and Yang, 2015; Alsbaiee et al., 2016; Li et al., 2017; Jie et al., 2018; Chen et al., 2019; Yang et al., 2019). Naphthalene-based macrocyclic arenes, termed as calixnaphthalenes, have also been produced (Poh et al., 1989; Andreetti et al., 1993; Shorthill et al., 2004; AlHujran et al., 2012; Avetta et al., 2012). However, calixnaphthalenes have not become highly popular receptors because they do not have unique molecular recognition properties. Considering that pillararenes with pillar-shape topologic structures have shown nice host-guest properties, we wondered whether we can create acyclic pillarnaphthalenes (Scheme 1), which would have deep, pillar-shape, and π-rich cavities, and maybe better binding abilities than calixnaphthalenes. As detailed below, we did not get such macrocycles, but succeed in making acyclic pillarnaphthalene oligomers.

Acyclic hosts that contain partially enclosed cavities capable of binding guests provided alternatives with unique synthetic and functional advantages (Goodman et al., 2007; Seebach and Gardiner, 2008; Pan et al., 2017; Wang et al., 2019a). For example, foldamers may provide cavities that are adaptive in recognizing different guest molecules (Zhang et al., 2012; Yashima et al., 2016). Molecular tweezers have made the way from a supramolecular host to a drug candidate, due to their ability to inhibit peptide and protein aggregation through the complexation toward amino acids (Sinha et al., 2011; Schrader et al., 2016).

Isaacs and his co-workers created acyclic cucurbit[*n*]uril-type receptors, which can function as solubilizing agents for insoluble drugs. Interestingly, the solubility of paclitaxel was increased 2,750 times through the formation of soluble container–drug complex (Ma et al., 2012). These highly soluble acyclic cucurbiturils could also solubilize individual single-walled carbon nanotubes (SWNTs) in water even at a concentration 100–1,000 times lower than typically required for surfactants (Shen et al., 2012). The groups of Schrader and Yoshizawa synthesized beautiful water-soluble clip and tweezer-shaped hosts based on norbornene and anthracene building blocks (Bier et al., 2013; Jono et al., 2017).

Herein, we wish to report the synthesis of a new type of receptors, acyclic pillar[*n*]naphthalene (*n* = 2–4, **Dimer**, **Trimer**, and **Tetramer**) oligomers, which are made up of 2,3-diethoxynaphthalene units linked by methylene bridges at the 1- and 4-positions. **Tetramer**, bearing a pseudo-cavity, has good host-guest properties toward a series of model organic cationic guests.

## Materials and Methods

All the reagents involved in this research were commercially available and used without further purification unless otherwise noted. ^1^H NMR, ^13^C NMR, 2D NOESY, and COSY spectra (see Supplementary Material) were recorded with a Bruker AVANCE III 500 MHz instrument. Chemical shifts were referred to TMS. Highresolution mass spectra (HRMS) were determined on a Bruker Daltonics, Inc. APEXIII 7.0 TESLA FTMS instrument. The single crystal X-ray data were measured by direct methods using SHELXS-971 and refined by fullmatrix least-squares procedures on F2 with SHELXL-97.2. All non-hydrogen atoms were obtained from the difference Fourier map and subjected to anisotropic refinement by full-matrix least squares on F2. Hydrogen atoms were obtained geometrically and treated as riding on the parent atoms or were constrained in the locations during refinements. Test parameters and detailed experimental data are shown in the Supplementary Material.

### Synthesis and Characterization

To the solution of 2,3-diethoxy naphthalene (2.6 g, 12 mmol) in CHCl_3_ (150 mL) was added paraformaldehyde (0.36 g, 12 mmol). Boron trifluoride diethyl etherate (2.5 ml, 20 mmol) was then added to the reaction mixture. The mixture was stirred at 25°C for 1 h. Then the reaction was quenched by addition of 50 mL water. The organic phase was separated and washed with saturated aqueous NaHCO_3_, and water. The organic layer was dried over anhydrous Na_2_SO_4_ and concentrated. The residue was purified by column chromatography on silica gel (eluent: 1/1, *v/v*, dichloromethane: petrol ether) to afford **Dimer** (21%), **Trimer** (9%), and **Tetramer** (15%), as white solids.

**Dimer**. m.p. 155–156°C. ^1^H NMR (500 MHz, CDCl_3_, 298 K): δ (ppm): 8.10 (d, *J* = 8.4 Hz, 2H), 7.61 (d, *J* = 7.7 Hz, 2H), 7.28–7.24 (m, 2H), 7.23–7.20 (m, 2H), 7.07 (s, 2H), 5.00 (s, 2H), 4.22 (q, *J* = 7.0 Hz, 4H), 4.02 (q, *J* = 7.0 Hz, 4H), 1.57 (t, *J* = 7.0 Hz, 6H), 1.33 (t, *J* = 7.0 Hz, 6H). ^13^C NMR (125 MHz, CDCl_3_, 298 K): δ (ppm): 151.36, 146.37, 131.43, 129.97, 128.57, 126.80, 124.88, 124.65, 123.71, 106.85 (C of acyclic dimer), 69.11, 63.80 (C of methylene in ethoxy group), 23.55 (C of methylene bridge of acyclic dimer), 15.58, 14.86 (C of methyl in ethoxy group). HRMS (ESI): C_29_H_32_O_4_${\text{NH}}_{4}^{+}$, calcd m/z 462.2644; found m/z 462.2641.

**Trimer**. m.p. 171–172°C. ^1^H NMR (500 MHz, CDCl_3_, 298 K): δ (ppm): 8.17 (d, *J* = 8.6 Hz, 2H), 8.04 (dd, *J* = 6.5, 3.3 Hz, 2H), 7.57 (d, *J* = 7.8 Hz, 2H), 7.23 (dd, *J* = 11.0, 4.0 Hz, 2H), 7.14–7.09 (m, 4H), 7.01 (s, 2H), 4.93 (s, 4H), 4.20 – 4.11 (m, 8H), 3.87 (q, *J* = 7.0 Hz, 4H), 1.51 (t, *J* = 7.0 Hz, 6H), 1.35 (t, *J* = 7.0 Hz, 6H), 1.13 (t, *J* = 7.0 Hz, 6H). ^13^C NMR (125 MHz, CDCl_3_, 298 K): δ (ppm): 151.37, 148.98, 146.30, 131.42, 130.70, 130.15, 128.93, 128.49, 126.81, 125.16, 124.90, 124.53, 124.39, 123.24, 106.78 (C of acyclic trimer), 69.21, 69.10, 63.79 (C of methylene in ethoxy group), 23.37 (C of methylene bridge of acyclic trimer), 15.77, 15.43, 14.84, 14.22 (C of methyl in ethoxy group). HRMS (ESI): C_44_H_48_O_6_${\text{NH}}_{4}^{+}$, calcd m/z 690.3795; found m/z 690.3786.

**Tetramer**. m.p. 212–213°C. ^1^H NMR (500 MHz, CDCl_3_, 298 K): δ (ppm): 8.19 (d, *J* = 8.2 Hz, 2H), 8.13 (d, *J* = 8.5 Hz, 2H), 8.02 (d, *J* = 8.2 Hz, 2H), 7.58 (d, *J* = 8.0 Hz, 2H), 7.23 (t, *J* = 7.6 Hz, 2H), 7.16–7.04 (m, 6H), 7.02 (s, 2H), 4.92 (s, 4H), 4.89 (s, 2H), 4.17 (q, *J* = 7.0 Hz, 4H), 4.13 (q, *J* = 7.0 Hz, 4H), 3.95 (q, *J* = 7.0 Hz, 4H), 3.88 (q, *J* = 7.0 Hz, 4H), 1.52 (t, *J* = 6.9 Hz, 6H), 1.33 (t, *J* = 7.0 Hz, 3H), 1.16 (t, *J* = 7.0 Hz, 6H), 1.05 (t, *J* = 7.0 Hz, 6H). ^13^C NMR (125 MHz, CDCl_3_, 298 K): δ (ppm): 151.36, 149.00, 148.89, 146.25, 131.42, 130.71, 130.66, 130.17, 129.29, 128.78, 128.46, 126.80, 125.21, 125.19, 124.91, 124.56, 124.29, 123.92, 123.18, 106.72 (C of acyclic tetramer), 69.25, 69.13, 69.08, 63.77 (C of methylene in ethoxy group), 23.34 (C of methylene bridge of acyclic tetramer), 15.78, 15.48, 15.46, 14.85 (C of methyl in ethoxy group). HRMS (ESI): C_59_H_64_O_8_${\text{NH}}_{4}^{+}$, calcd m/z 918.4945; found m/z 918.4922.

## Results and Discussion

2,3-Diethoxy naphthalene was selected as the building block to condense with paraformaldehyde. Due to the electron-donating ethoxy groups, great regioselectivity can be rationalized, and the reactive sites should be 1- and 4-positions in Friedel–Crafts reaction. It was expected to produce pillar-shape macrocycles, pillar[*n*]naphthalenes. However, no cyclic oligomers have been obtained after many attempts; a possible reason is that big naphthalene units make the final cyclization quite difficult due to the steric hindrance. Fortunately, we got acyclic pillar[*n*]naphthalenes (*n* = 2–4).

**Scheme 2:**
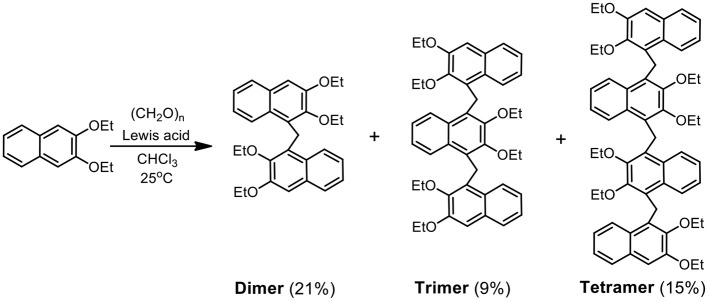
Synthesis of acyclic pillar[*n*]naphthalenes **Dimer**, **Trimer**, and **Tetramer**.

Using BF_3_·(Et)_2_O as the catalyst, the condensation reaction of 2,3-diethoxy naphthalene and paraformaldehyde in CHCl_3_ at room temperature (Scheme 2) produced oligomers **Dimer**, **Trimer**, and **Tetramer** with yields of 21, 9, and 15%, respectively. Other Lewis acid catalysts, for example TfOH, FeCl_3_, and AlCl_3_, could also work, but the reaction yields were lower than that for BF_3_·(Et)_2_O. The synthesis was considerably easy since it just involved a one–step reaction of commercial starting materials and the isolation was also convenient by column chromatography on silica gel.

**Dimer**, **Trimer**, and **Tetramer** were well characterized by ^1^H NMR, ^13^C NMR, NOESY, and COSY spectra (Figure 1 and Supplementary Figures 1–11), and high-resolution mass spectrometry (HRMS). They have rather complex patterns of aromatic and ethoxy peaks in ^1^H NMR spectra (Figure 1) because they are not highly symmetrical macrocycles, but acyclic oligomers with low symmetry.

**Figure 1:**
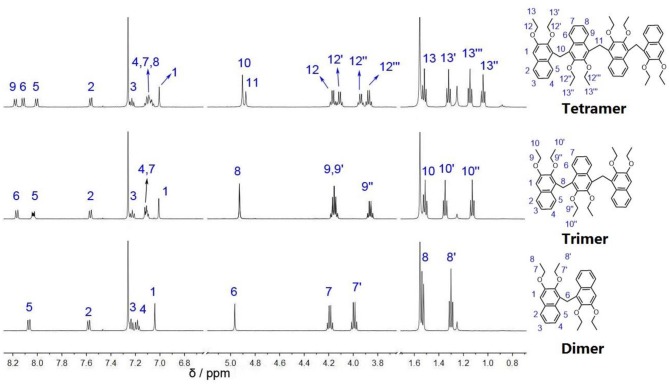
^1^H NMR spectra (500 MHz, 2.0 mM, CDCl_3_) of **Dimer**, **Trimer**, and **Tetramer**.

**Figure 2:**
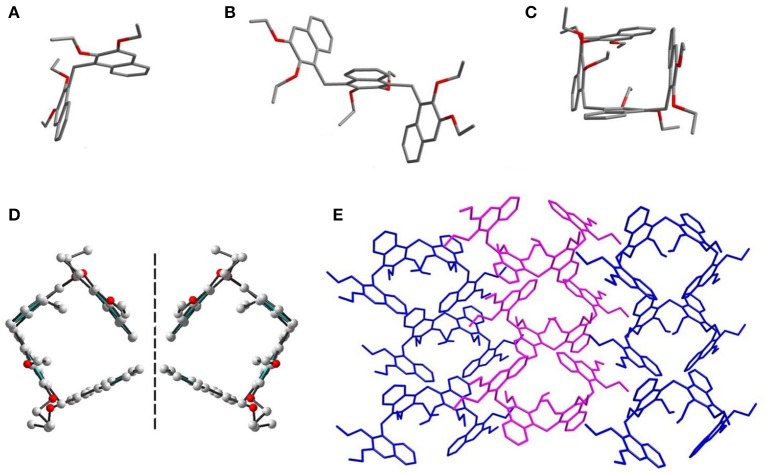
Crystal structures of **Dimer (A)**, **Trimer (B)**, and **Tetramer (C)**. **(D)** A pair of enantiomers of **Tetramer**. **(E)** Packing mode of **Tetramer**.

Single crystals of **Dimer, Trimer**, and **Tetramer** suitable for X-ray analysis were obtained by diffusion of hexane into a solution of the compounds in dichloromethane at room temperature (Figure 2). As expected, these three acyclic oligomers had the same connecting style, i.e., 2,3-diethoxy naphthalene units were connected by methylene at 1,4-positions. As shown in Figures 2A,B, the acyclic **Dimer** and **Trimer**, possessing two and three naphthalene moieties, have ill–defined cavities. Particularly, the **Tetramer** exhibits a pseudocycle–shaped structure, with all the methylene bridges being orientated outwardly. There exist intramolecular sextuple C–H···π interactions, with H···ring center distances of 2.75–3.23 Å (Supplementary Figure 12), between the middle two ethoxy groups and naphthalenes, resulting in the formation of a pseudo cycle rather than a zigzag structure. More interestingly, the single crystal structures of **Tetramer** molecules exist in a pair of enantiomers in the solid state (Figure 2D).

**Scheme 3:**
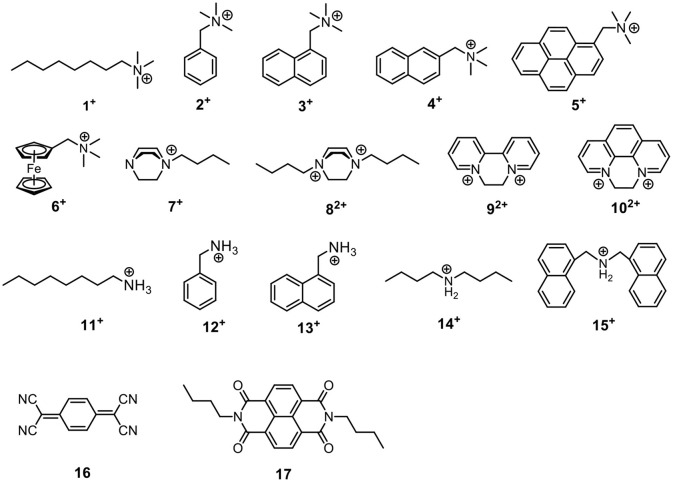
Structures of guest molecules. The counter anions of **1**^+^-**15**^+^ are tetrakis[3,5-bis(trifluoromethyl)phenyl] borate (BArF^−^).

The host-guest properties of the acyclic receptors were then tested. Since they possess π-rich cavities, several cationic guests (**1**^+^-**15**^+^) and electron–deficient neutral guests (**16**–**17**) (Scheme 3) were chosen as model guest molecules to investigate their host-guest chemistry. In most cases, CDCl_3_ was used as solvent during the ^1^H NMR experiments of host-guest mixture and following NMR titrations; for guests **7**^**2+**^**, 9**^**2+**^, and **10**^**2+**^, CD_2_Cl_2_ was used because of their poor solubility in CDCl_3_.

**Figure 3:**
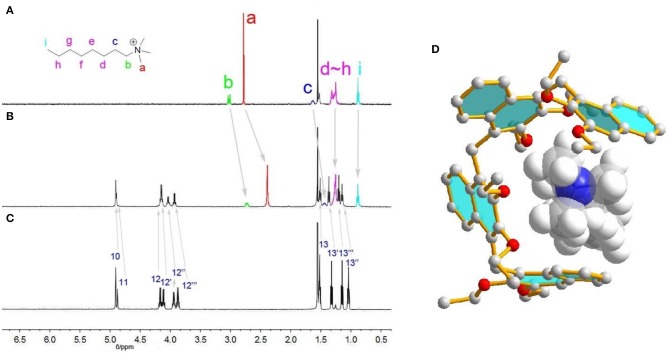
^1^H NMR spectra (CDCl_3_, 298 K, 1.0 mmol) of **(A)** guest **1**^**+**^, **(B) 1**^**+**^ and **Tetramer** (1:1 mixture), **(C) Tetramer**. **(D)** Energy-minimized structures of **1**^**+**^•**Tetramer** at the semiempirical PM6 level of theory.

Figure 3 shows the ^1^H NMR spectra recorded for quarternary ammonium guest **1**^**+**^ in the absence and presence of **Tetramer**. As can be readily seen, upon addition of **Tetramer**, protons H_a_, H_b_ and H_c_ of **1**^**+**^ display substantial upfield shifts (Δδ = −0.39, −0.29, and −0.21 ppm) due to complexation–induced shielding effects, indicating that **1**^**+**^ was located inside the acyclic host's pseudo-cavity to form a host-guest inclusion complex, and the main binding site is the N^+^(Me)_3_ moiety. In contrast, protons H_h−i_ undergo indistinct NMR changes, suggesting they are located outside the cavity of **Tetramer**. [24] In the NOESY spectrum of **1**^**+**^ and **Tetramer**, NOE correlations were observed between methyl protons H_a_ of the guest and the aromatic protons H_5_, H_7_ and H_8_ of **Tetramer**, also suggesting the host-guest encapsulation (Supplementary Figure 13). The formation of **1**^**+**^•**Tetramer** complex was further supported by ESI mass spectrometry analysis of an equimolar mixture of **1**•BArF and **Tetramer**, where an intense peak for the 1:1 complex (*m*/*z* 1072.66, calcd. for **1**^**+**^•**Tetramer** = 1072.67) was observed (Supplementary Figure 14). The encapsulation could also be rationalized by energy-minimized molecular modeling (Figure 3D): the oligomers wrapped around the guest to enhance the host-guest contacts driven by cation···π/ C–H···π interactions.

The addition of **Dimer** and **Trimer** could also induce the upfield shifts of guest **1**^**+**^, but the Δδ values are smaller than those for **Tetramer** (Supplementary Figure 15). These results indicated relatively weak binding interactions occurred for **Dimer** and **Trimer** in comparison with **Tetramer**. These observations were consistent with the association constants (*K*_a_) obtained from ^1^H NMR titration experiments. As shown in Table 1, the *K*_a_ value of **1**^+^ with **Tetramer** [(4.4±0.6) × 10^2^ M^−1^] is 18 times larger than that for **Trimer**, and the affinity for **Dimer** was too small to be accurately calculated (< 5 M^−1^).

**Table 1:** Association constants (M^−1^) of **Dimer**, **Trimer**, and **Tetramer** with different guests (500 MHz, 298 K).

**Guest**	**Host**	**Solvent**	***K*^a^ (M^**−1**^)^a^**
**1**^**+**^	**Dimer**	CDCl_3_	_–_^b^
**1**^**+**^	**Trimer**	CDCl_3_	25 ± 7
**1**^**+**^	**Tetramer**	CDCl_3_	(4.4 ± 0.6) × 10^2^
**2**^**+**^	**Tetramer**	CDCl_3_	(2.9 ± 0.4) × 10^2^
**3**^**+**^	**Tetramer**	CDCl_3_	(1.2 ± 0.2) × 10^3^
**4**^**+**^	**Tetramer**	CDCl_3_	(2.1 ± 0.4) × 10^3^
**5**^**+**^	**Tetramer**	CDCl_3_	(1.6 ± 0.2) × 10^2^
**6**^**+**^	**Tetramer**	CDCl_3_	(1.8 ± 0.2) × 10^2^
**7**^2+^	**Tetramer**	CDCl_3_	(2.0 ± 0.1) × 10^2^
**8**^**+**^	**Tetramer**	CD_2_Cl_2_	(1.4 ± 0.1) × 10^2^
**9**^2+^	**Tetramer**	CD_2_Cl_2_	(1.2 ± 0.2) × 10^2^
**10**^2+^	**Tetramer**	CD_2_Cl_2_	(1.7 ± 0.3) × 10^2^
**11**^**+**^	**Tetramer**	CDCl_3_	(2.5 ± 0.4) × 10^4^
**12**^**+**^	**Tetramer**	CDCl_3_	(4.3 ± 0.3) × 10^3^
**13**^**+**^	**Tetramer**	CDCl_3_	(1.4 ± 0.1) × 10^4^
**14**^**+**^	**Tetramer**	CDCl_3_	(1.4 ± 0.2) × 10^3^
**15**^**+**^	**Tetramer**	CDCl_3_	(3.0 ± 0.3) × 10^2^
**16–17**	**Tetramer**	CDCl_3_	_—_^c^

a *The K_a_ values were determined by NMR titrations (Supplementary Figure 30)*.

b *The K_a_ value was too small (<5 M^−1^) to be accurately calculated*.

c *No interactions were found (Supplementary Figures 28, 29)*.

Since **Tetramer** showed interesting structure and good recognition behavior, we then examined its binding capacity toward other cationic guests (Table 1 and Supplementary Figures 16–29), revealing that **Tetramer** can form host–guest complexes with them but the binding affinities are totally different. For the trimethyl ammonium guests **1**^+^-**6**^+^, **3**^**+**^ [*K*_a_ = (1.2 ± 0.2) × 10^3^ M^−1^] and **4**^**+**^ [*K*_a_ = (2.1±0.4) × 10^3^ M^−1^] bearing naphthyl moieties give stronger affinities, which should be due to host-guest fitted π ··· π interactions and large contacts. The substitution of naphthyl for smaller phenyl or bigger pyrenyl in **3**^**+**^ and **4**^**+**^, affording **1**^**+**^ or **5**^**+**^, considerably decreases the association constants by one order of magnitude.

Binding affinities of **Tetramer** toward primary ammonium guests **11**^**+**^–**13**^**+**^ were stronger than those of the corresponding quaternary ammonium guests **1**^**+**^–**3**^**+**^. For example, the *K*_a_ value of **Tetramer** and octylammonium **11**^**+**^ [(2.5±0.4) × 10^4^ M^−1^] is about 56-fold higher than that for trimethyloctylammonium **1**^**+**^ [(4.4±0.6) × 10^2^ M^−1^]. Similarly, the selectivity factors of **12**^**+**^/**2**^**+**^ and **13**^**+**^/**2**^**+**^ are 15 and 12, respectively. The reason for such high selectivity would be that big and spherical N^+^(Me)_3_ group is too larger compared with **Tetramer**'s size, and small ${\text{NH}}_{3}^{+}$ is a suitable one. It should be noted that the binding affinities of **Tetramer** and organic ammonium salts, with *K*_a_ values in the magnitude of 10^2^-10^4^ M^−1^, are comparable to those for macrocyclic arenes such as pillararenes and biphenarenes.

Due to its π-electron rich cavity, the complexation of **Tetramer** and two π-deficient neutral guests, **16** and **17**, were also investigated. From Supplementary Figures 28, 29, no obvious NMR changes were detected, indicating no stable complexes can be formed.

## Conclusions

In summary, acyclic pillarnaphthalenes with 2,3-diethoxynaphthalene units bridged by methylenes at 1,4-positions were synthesized through a one-pot reaction of 2,3-diethoxy naphthalene monomer and paraformaldehyde by using Lewis acid as the catalyst. Acyclic pillar[4]naphthalene **Tetramer** is able to interact organic ammonium guests cations by wrapping around them, giving association constants in the magnitude of 10^2^-10^4^ M^−1^. We expect that **Tetramer** bearing pseudo-cycle cavity, could have significant potential for the applications in host-guest chemistry and self-assembly.

## Data Availability Statement

All datasets generated for this study are included in the article/Supplementary Material.

## Author Contributions

CL, BW, and YJ conceived this project and designed the experiments. YJ and MD contributed to most of the experimental work. CL, MD, and BW co-wrote the paper. All authors discussed and commented on the paper and analyzed the data.

### Conflict of Interest

The authors declare that the research was conducted in the absence of any commercial or financial relationships that could be construed as a potential conflict of interest.

## References

[B1] AlHujran T. A. Dawe L. N. Georghiou P. E. (2012). Synthesis of functionalized acenaphthenes and a new class of homooxacalixarenes. Org. Lett. 14, 3530–3533. 10.1021/ol301538s 22724527

[B2] Alsbaiee A. Smith B. J. Xiao L. Ling Y. Helbling D. E. Dichtel W. R. (2016). Rapid removal of organic micropollutants from water by a porous β-cyclodextrin polymer. Nature 529, 190–194. 10.1038/nature16185 26689365

[B3] Andreetti G. D. Boehmer V. Jordon J. G. Tabatabai M. Ugozzoli F. Vogt W. (1993). Dissymmetric calix [4] arenes with C4-and C2-symmetry. Synthesis, X-ray structures, conformational fixation, and proton NMR spectroscopic studies. J. Org. Chem. 58, 4023–4032. 10.1021/jo00067a040

[B4] Avetta C. T. Shorthill B. J. Ren C. Glass T. E. (2012). Molecular tubes for lipid sensing: tube conformations control analyte selectivity and fluorescent response. J. Org. Chem. 77, 851–857. 10.1021/jo201791a 22263717

[B5] Baldini L. Casnati A. Sansone F. Ungaro R. (2007). Calixarene-based multivalent ligands. Chem. Soc. Rev. 36, 254–266. 10.1039/B603082N 17264928

[B6] Bier D. Rose R. Bravo-Rodriguez K. Bartel M. Ramirez-Anguita J. M. Dutt S.. (2013). Molecular tweezers modulate 14-3-3 protein–protein interactions. Nat. Chem. 5, 234–239. 10.1038/nchem.1570 23422566

[B7] Chen H. Fan J. Hu X. Ma J. Wang S. Li J.. (2015). Biphen[n]arenes. Chem. Sci. 6, 197–202. 10.1039/C4SC02422B 28553468 PMC5424672

[B8] Chen J. Ni H. Meng Z. Wang J. Huang X. Dong Y.. (2019). Supramolecular trap for catching polyamines in cells as an anti-tumor strategy. Nat. Commun. 10:3546. 10.1038/s41467-019-11553-7 31391464 PMC6685945

[B9] Chun Y. Singh N. J. Hwang I. C. Lee J. W. Yu S. U. Kim K. S. (2013). Calix [n] imidazolium as a new class of positively charged homo-calix compounds. Nat. Commun. 4:1797. 10.1038/ncomms2758 23653209 PMC3644089

[B10] Cram D. J. (1988). The design of molecular hosts, guests, and their complexes. Science 240, 760–767. 10.1126/science.3283937 3283937

[B11] Dai L. Ding Z. J. Cui L. Li J. Jia X. Li C. (2017). 2, 2′-Biphen[n]arenes (n = 4–8): one-step, high-yield synthesis, and host–guest properties. Chem. Commun. 53, 12096–12099. 10.1039/C7CC06767D 29071314

[B12] Gong H. Y. Rambo B. M. Karnas E. Lynch V. M. Sessler J. L. (2010). A ‘Texas-sized'molecular box that forms an anion-induced supramolecular necklace. Nat. Chem. 2, 406–409. 10.1038/nchem.597 20414243

[B13] Goodman C. M. Choi S. Shandler S. DeGrado W. F. (2007). Foldamers as versatile frameworks for the design and evolution of function. Nat. Chem. Biol. 3, 252–262. 10.1038/nchembio876 17438550 PMC3810020

[B14] Guo D. S. Liu Y. (2012). Calixarene-based supramolecular polymerization in solution. Chem. Soc. Rev. 41, 5907–5921. 10.1039/C2CS35075K 22617955

[B15] Guo S. Song Y. He Y. Hu X. Y. Wang L. (2018). Highly efficient artificial light-harvesting systems constructed in aqueous solution based on supramolecular self-assembly. Angew. Chem. Int. Ed. 57, 3163–3167. 10.1002/anie.201800175 29383817

[B16] Jie K. Zhou Y. Li E. Huang F. (2018). Nonporous adaptive crystals of pillararenes. Acc. Chem. Res. 51, 2064–2072. 10.1021/acs.accounts.8b00255 30011181

[B17] Jono K. Suzuki A. Akita M. Albrecht K. Yamamoto K. Yoshizawa M. (2017). A polyaromatic molecular clip that enables the binding of planar, tubular, and dendritic compounds. Angew. Chem. Int. Ed. 129, 3570–3574. 10.1002/anie.201612489 28225169

[B18] Jurícek M. Strutt N. L. Barnes J. C. Butterfield A. M. Dale E. J. Baldridge K. K.. (2014). Induced-fit catalysis of corannulene bowl-to-bowl inversion. Nat. Chem. 6, 222–228. 10.1038/nchem.1842 24557137

[B19] Lehn J. M. (1988). Supramolecular chemistry—scope and perspectives molecules, supermolecules, and molecular devices (Nobel Lecture). Angew. Chem. Int. Ed. 27, 89–112. 10.1002/anie.198800891

[B20] Li B. Meng Z. Li Q. Huang X. Kang Z. Dong H.. (2017). A pH responsive complexation-based drug delivery system for oxaliplatin. Chem. Sci. 8, 4458–4464. 10.1039/c7sc01438d 28970876 PMC5618340

[B21] Li B. Wang B. Huang X. Dai L. Cui L. Li J.. (2019). Terphen[n]arenes and quaterphen[n]arenes (n = 3–6): one-pot synthesis, self-assembly into supramolecular gels, and iodine capture. Angew. Chem. Int. Ed. 58, 3885–3889. 10.1002/anie.201813972 30600896

[B22] Liu Y. Zhao W. Chen C. H. Flood A. H. (2019). Chloride capture using a C–H hydrogen bonding cage. Science 365, 159–161. 10.1126/science.aaw5145 31123106

[B23] Luo J. Ao Y. F. Wang Q. Q. Wang D. X. (2018). Diversity-oriented construction and interconversion of multicavity supermacrocycles for cooperative anion–π binding. Angew. Chem. Int. Ed. 57, 15827–15831. 10.1002/anie.201810836 30295403

[B24] Ma D. Hettiarachchi G. Nguyen D. Zhang B. Wittenberg J. B. Zavalij P. Y.. (2012). Acyclic cucurbit [n] uril molecular containers enhance the solubility and bioactivity of poorly soluble pharmaceuticals. Nat. Chem. 4, 503–510. 10.1038/nchem.1326 22614387

[B25] Ma Y. L. Ke H. Valkonen A. Rissanen K. Jiang W. (2018). Achieving strong positive cooperativity through activating weak non-covalent interactions. Angew. Chem. Int. Ed. 57, 709–713. 10.1002/anie.201711077 29139184

[B26] Ogoshi T. Kanai S. Fujinami S. Yamagishi T. A. Nakamoto Y. (2008). para-Bridged symmetrical pillar [5] arenes: their Lewis acid catalyzed synthesis and host–guest property. J. Am. Chem. Soc. 130, 5022–5023. 10.1021/ja711260m 18357989

[B27] Pan S. J. Ye G. Jia F. He Z. Ke H. Yao H.. (2017). Regioselective synthesis of methylene-bridged naphthalene oligomers and their host–guest chemistry. J. Org. Chem. 82, 9570–9575. 10.1021/acs.joc.7b01579 28836436

[B28] Pedersen C. J. (1988). The discovery of crown ethers. Science 241, 536–540. 10.1126/science.241.4865.536 17774576

[B29] Poh B. L. Lim C. S. Khoo K. S. (1989). A water-soluble cyclic tetramer from reacting chromotropic acid with formaldehyde. Tetrahedron Lett. 30, 1005–1008. 10.1016/S0040-4039(00)95302-4

[B30] Schrader T. Bitan G. Klärner F. G. (2016). Molecular tweezers for lysine and arginine–powerful inhibitors of pathologic protein aggregation. Chem. Commun. 52, 11318–11334. 10.1039/C6CC04640A 27546596 PMC5026632

[B31] Seebach D. Gardiner J. (2008). β-peptidic peptidomimetics. Acc. Chem. Res. 41, 1366–1375. 10.1021/ar700263g 18578513

[B32] Shen C. Ma D. Meany B. Isaacs L. Wang Y. (2012). Acyclic cucurbit [n] uril molecular containers selectively solubilize single–walled carbon nanotubes in water. J. Am. Chem. Soc. 134, 7254–7257. 10.1021/ja301462e 22512431

[B33] Shorthill B. J. Avetta C. T. Glass T. E. (2004). Shape-selective sensing of lipids in aqueous solution by a designed fluorescent molecular tube. J. Am. Chem. Soc. 126, 12732–12733. 10.1021/ja047639d 15469241

[B34] Sinha S. Lopes D. H. Du Z. Pang E. S. Shanmugam A. Lomakin A.. (2011). Lysine-specific molecular tweezers are broad-spectrum inhibitors of assembly and toxicity of amyloid proteins. J. Am. Chem. Soc. 133, 16958–16969. 10.1021/ja206279b 21916458 PMC3210512

[B35] Song N. Yang Y. W. (2015). Molecular and supramolecular switches on mesoporous silica nanoparticles. Chem. Soc. Rev. 44, 3474–3504. 10.1039/C5CS00243E 25904466

[B36] Wang M. X. (2018). Coronarenes: recent advances and perspectives on macrocyclic and supramolecular chemistry. Sci. China Chem. 61, 993–1003. 10.1007/s11426-018-9328-8

[B37] Wang Y. Liu T. Jiang J. Chen Y. Cen M. Lu D.. (2019a). Syntheses of water-soluble acyclic naphthalene oligomers and their applications in water. Dalton Trans. 48, 6333–6336. 10.1039/C9DT00709A 30973550

[B38] Wang Y. Ping G. Li C. (2016). Efficient complexation between pillar [5] arenes and neutral guests: from host–guest chemistry to functional materials. Chem. Commun. 52, 9858–9872. 10.1039/C6CC03999E 27351168

[B39] Wang Y. Xu K. Li B. Cui L. Li J. Jia X.. (2019b). Efficient separation of cis-and trans-1, 2-dichloroethene isomers by adaptive biphen[3]arene crystals. Angew. Chem. Int. Ed. 58, 10281–10284. 10.1002/anie.201905563 31112359

[B40] Xue M. I. N. Yang Y. Chi X. Zhang Z. Huang F. (2012). Pillararenes, a new class of macrocycles for supramolecular chemistry. Acc. Chem. Res. 45, 1294–1308. 10.1021/ar2003418 22551015

[B41] Yang B. Zhang X. D. Li J. Tian J. Wu Y. P. Yu F. X. (2019). *In situ* loading and delivery of short single-and double-stranded dna by supramolecular organic frameworks. CCS Chem. 1, 156–165 10.31635/ccschem.019.20180011

[B42] Yang K. Pei Y. Wen J. Pei Z. (2016). Recent advances in pillar [n] arenes: synthesis and applications based on host–guest interactions. Chem. Commun. 52, 9316–9326. 10.1039/C6CC03641D 27332040

[B43] Yashima E. Ousaka N. Taura D. Shimomura K. Ikai T. Maeda K. (2016). Supramolecular helical systems: helical assemblies of small molecules, foldamers, and polymers with chiral amplification and their functions. Chem. Rev. 116, 13752–13990. 10.1021/acs.chemrev.6b00354 27754649

[B44] Zhang D. W. Zhao X. Hou J. L. Li Z. T. (2012). Aromatic amide foldamers: structures, properties, and functions. Chem. Rev. 112, 5271–5316. 10.1021/cr300116k 22871167

[B45] Zhang G. W. Li P. F. Meng Z. Wang H. X. Han Y. Chen C. F. (2016). Triptycene-based chiral macrocyclic hosts for highly enantioselective recognition of chiral guests containing a trimethylamino group. Angew. Chem. Int. Ed. 55, 5304–5308. 10.1002/anie.201600911 27011062

