# Supplementary material for: Synthesis and Host–Guest Properties of Acyclic Pillar[n]naphthalenes
Source: Front Chem. 2019 Dec 3;7:828. doi: 10.3389/fchem.2019.00828 (PMC6901500; doi:10.3389/fchem.2019.00828)
Supplement: Supplementary file 1 [file Data_Sheet_1.DOCX]

**Supplementary Material**

Synthesis and Host−guest Properties of Acyclic Pillar[*n*]naphthalenes

Yuanyin Jia^1+^, Ming Dong^2+^, Bin Wang^2^*, Chunju Li^1,2,3^*

^1^ School of Chemical and Environmental Engineering, Shanghai Institute of Technology, Shanghai, 201418, P.R. China.

^2^ Key Laboratory of Inorganic-Organic Hybrid Functional Material Chemistry, Ministry of Education, Tianjin Key Laboratory of Structure and Performance for Functional Molecules, College of Chemistry, Tianjin Normal University, Tianjin, 300387, P. R. China.

^3^ Center for Supramolecular Chemistry and Catalysis and Department of Chemistry, Shanghai University, Shanghai, 200444, P. R. China.

^+^ These authors contributed equally to this work.

Email: hxxywangb@mail.tjnu.edu.cn, cjli@shu.edu.cn

**Table of Contents**

1. NMR spectra of **Dimer, Trimer and Tetramer…………………………………**2

2. X-ray crystal data of oligomers **Dimer, Trimer and Tetramer…………………**8

3. NOESY spectrum of **1**^+^•**Tetramer………………………………………………**9

3. Mass spectrum of **1**^+^•**Tetramer** **…………………………………………………**10

4. Additional ^1^H NMR spectra of host-guest mixture**……………………………**11

5. Determination of the association constants**……………………………………**19

6. Computational Data **………………………………………………………**20

7. References**………………………………………………………………………**24

1. NMR spectra of **Dimer, Trimer and Tetramer**


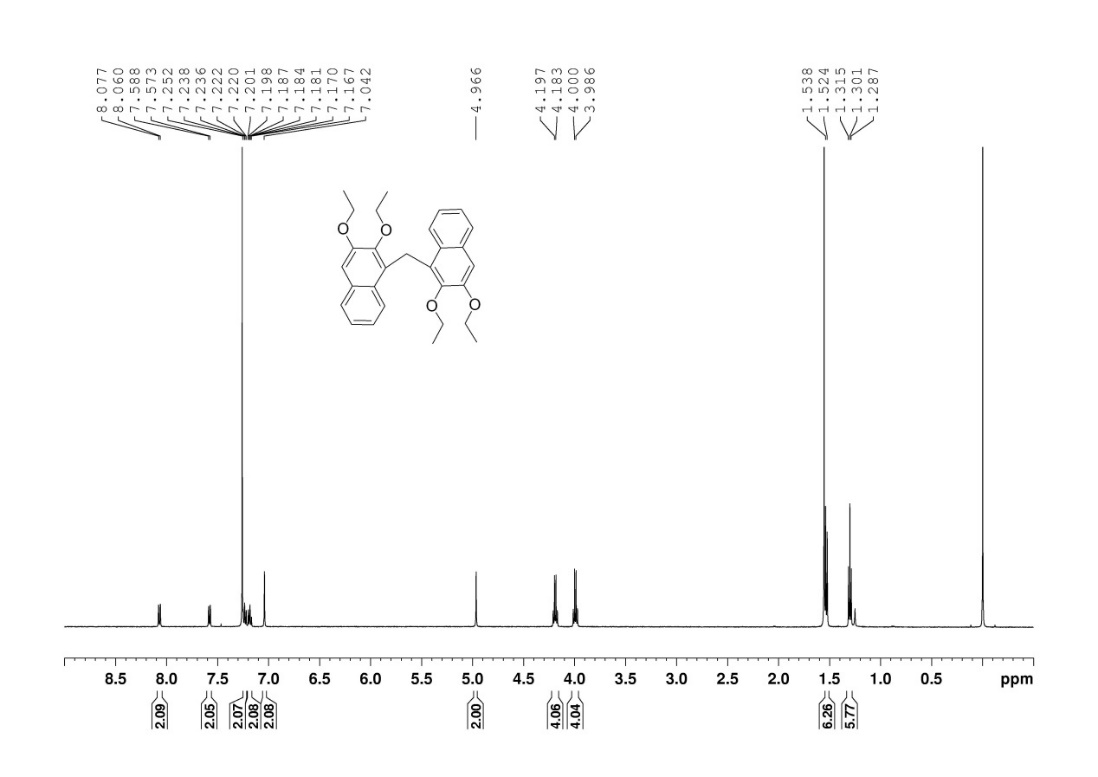


**Supplementary Figure 1.** ^1^H NMR spectrum (500 MHz, CDCl_3_, 298K) of **Dimer**


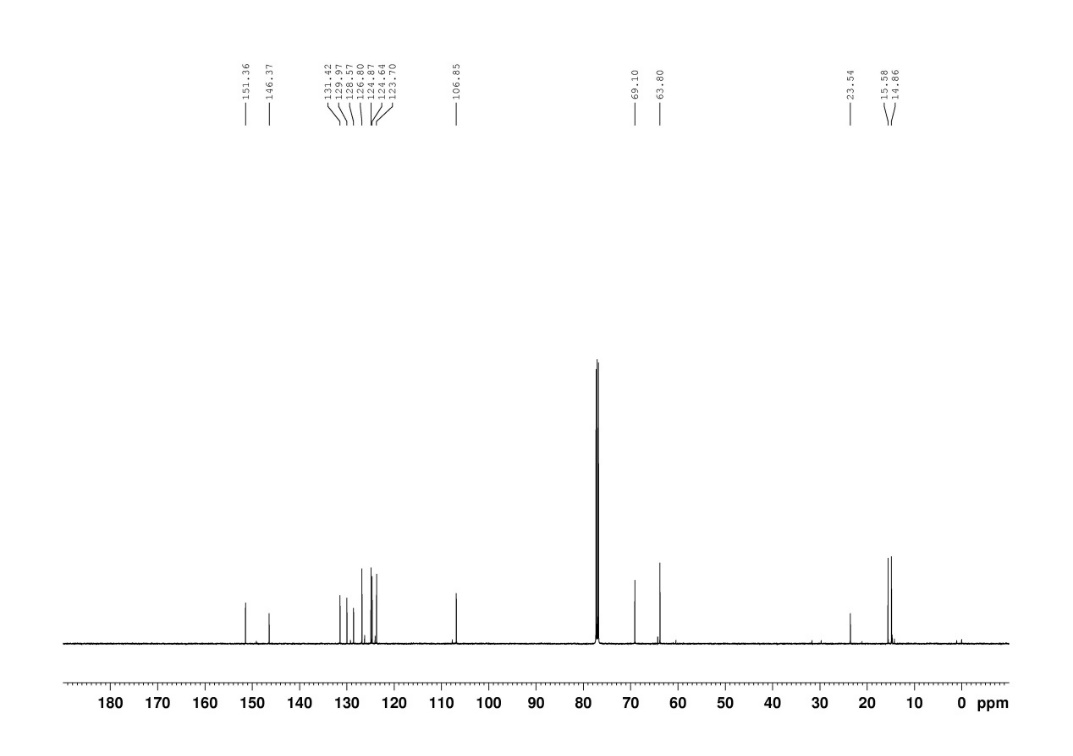


**Supplementary Figure 2.** ^13^C NMR spectrum (125 MHz, CDCl_3_, 298K) of **Dimer**


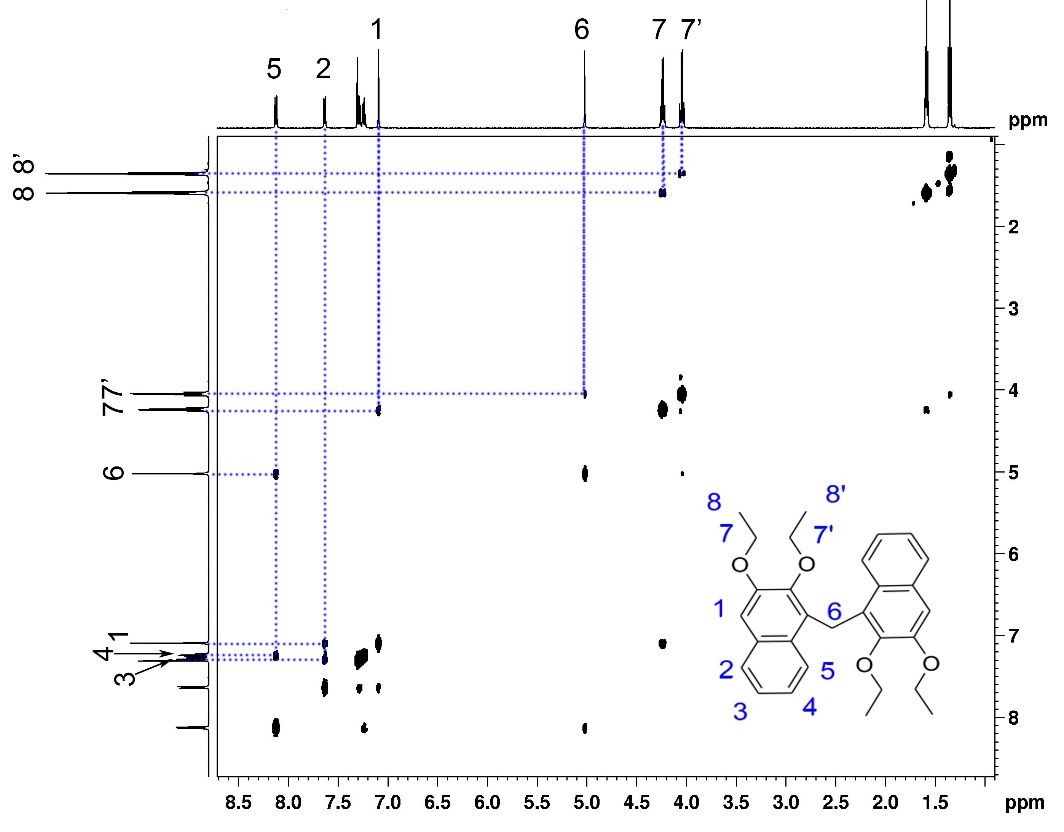


**Supplementary Figure 3.** 2D ^1^H,^1^H-NOESY spectrum (500 MHz, CDCl_3_, 298K) of **Dimer**


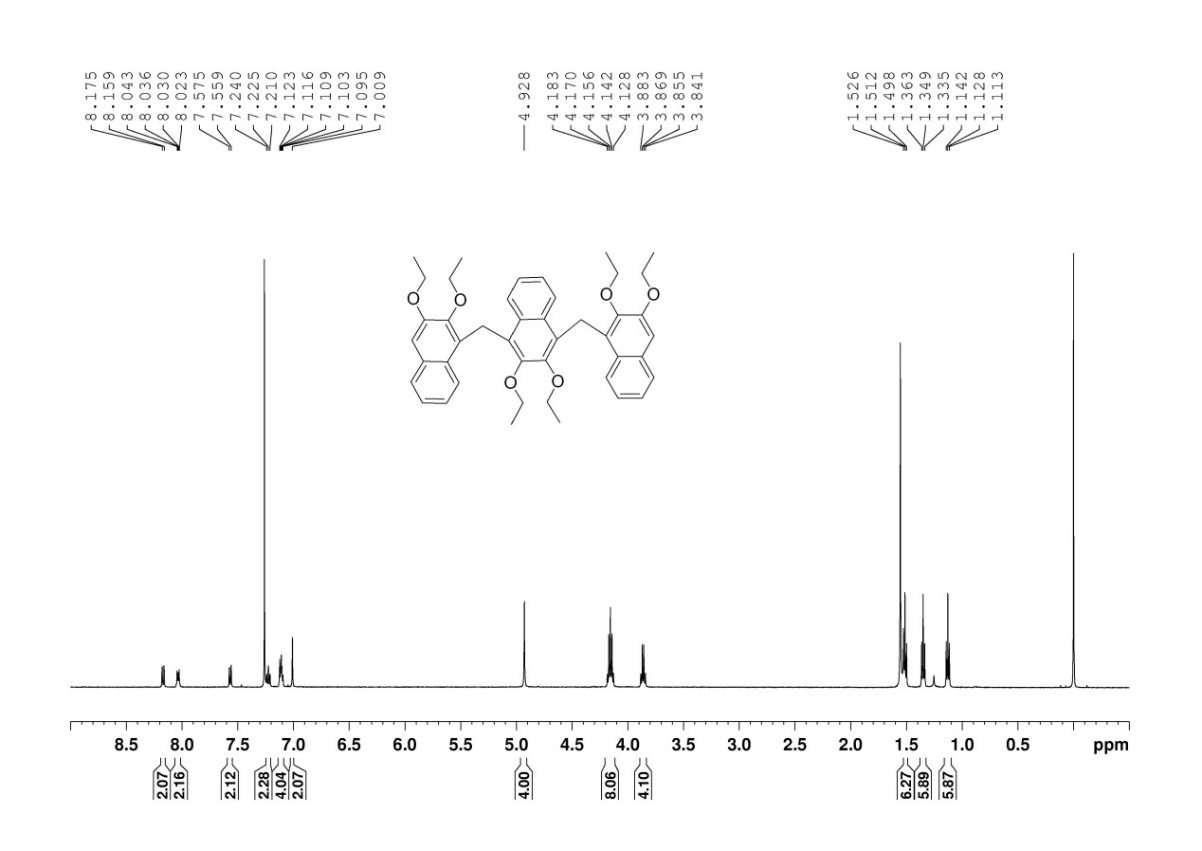


**Supplementary Figure 4.** ^1^H NMR spectrum (500 MHz, CDCl_3_, 298K) of **Trimer**


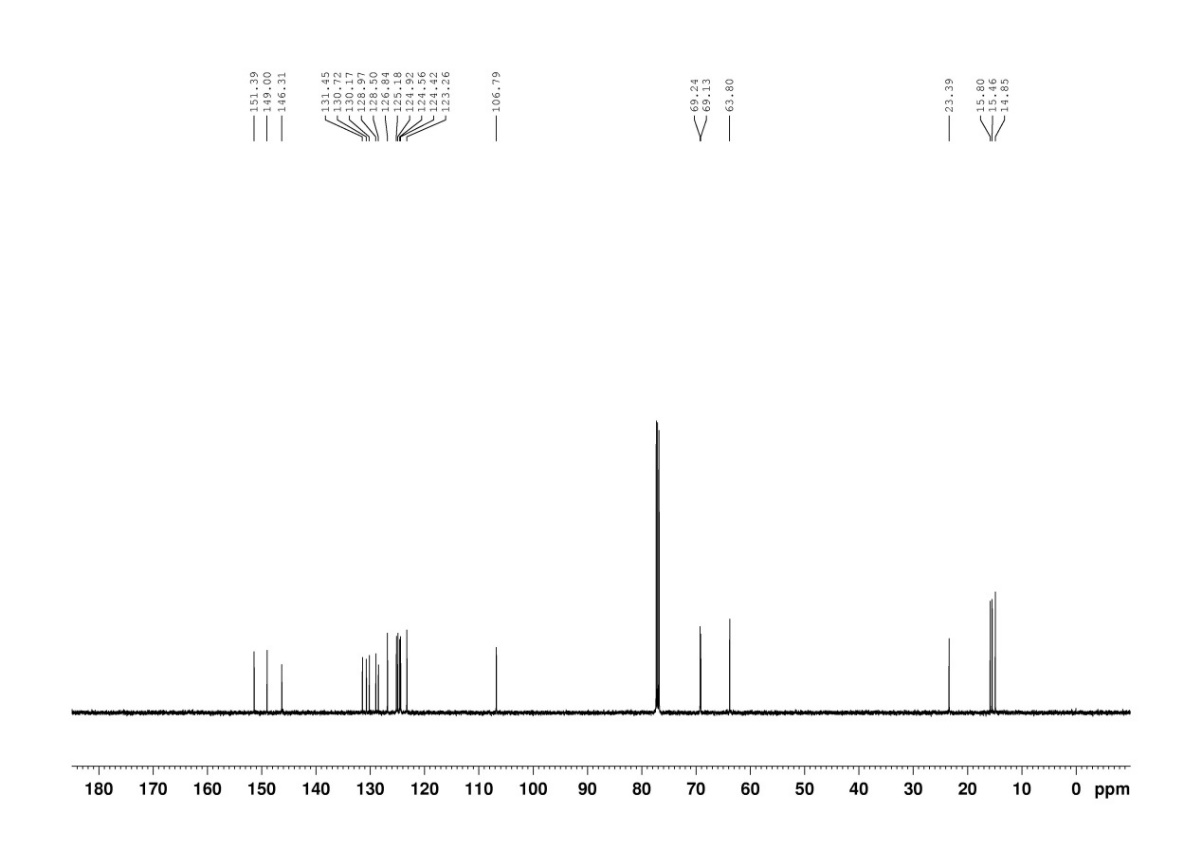


**Supplementary Figure 5.** ^13^C NMR spectrum (125 MHz, CDCl_3_, 298K) of **Trimer**


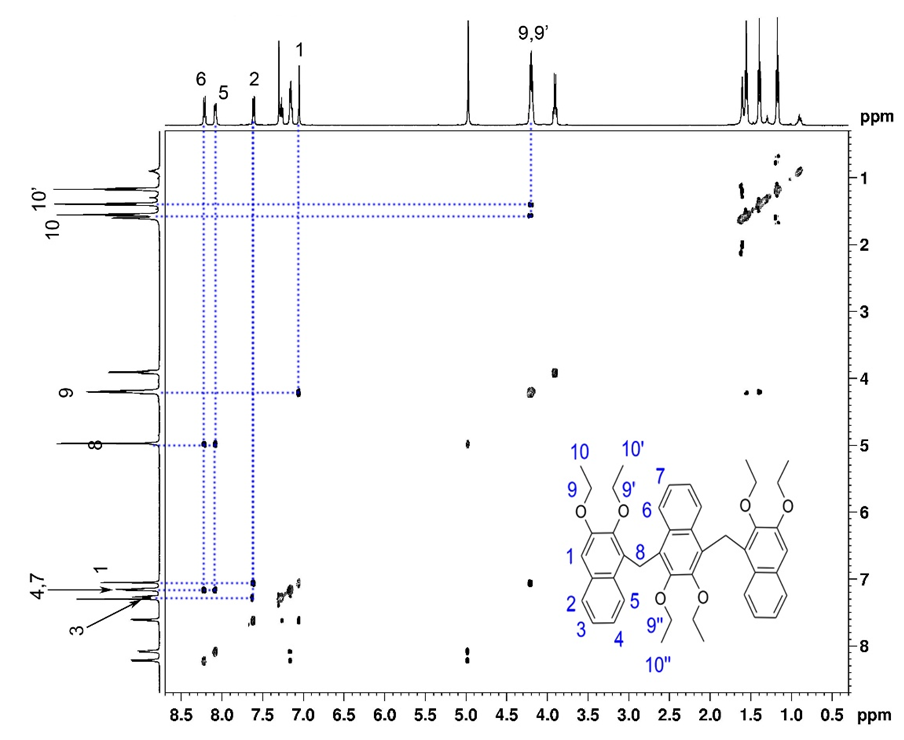


**Supplementary Figure 6.** 2D ^1^H,^1^H-NOESY spectrum (500 MHz, CDCl_3_, 298K) of **Trimer**


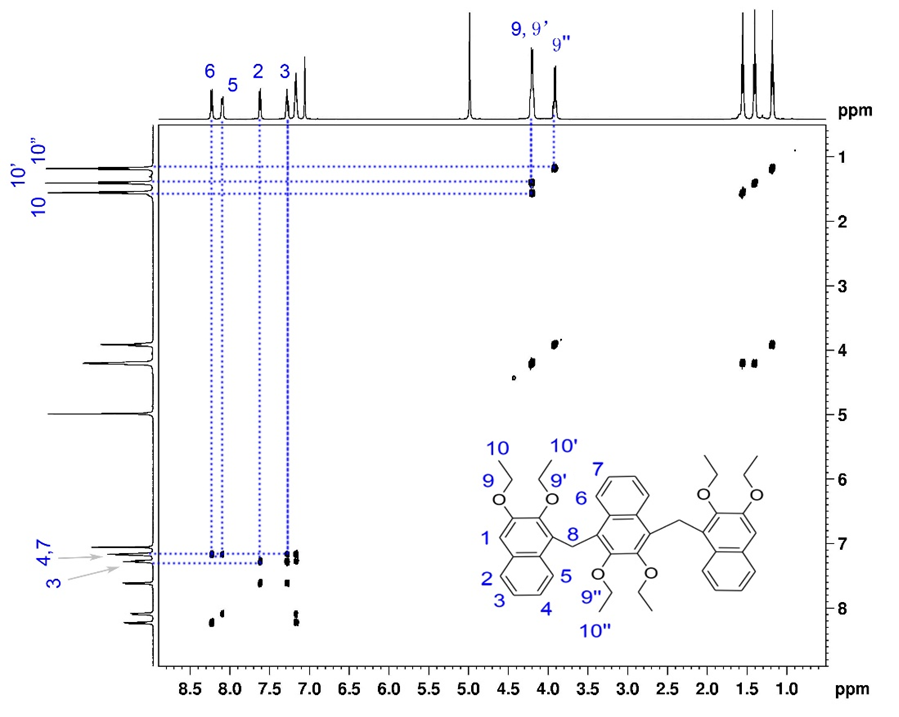


**Supplementary Figure 7.** 2D ^1^H,^1^H-COSY (500 MHz, CDCl_3_, 298K) of **Trimer**


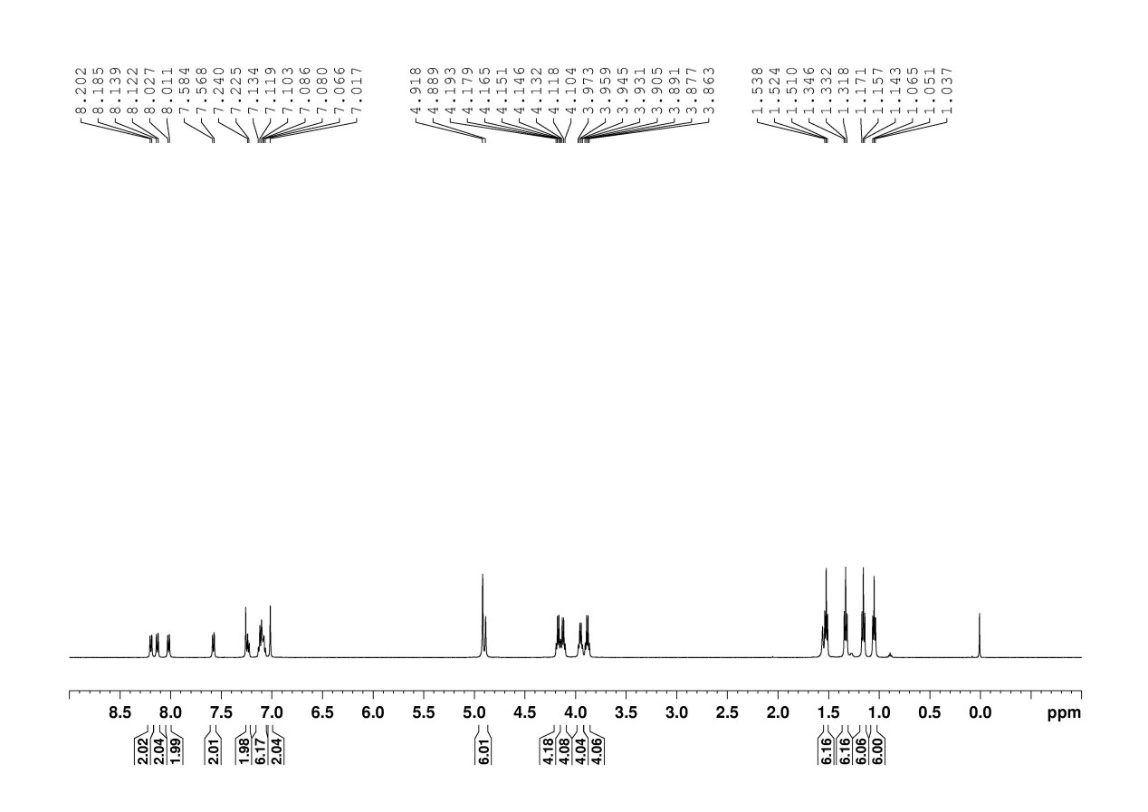


**Supplementary Figure 8.** ^1^H NMR spectrum (500 MHz, CDCl_3_, 298K) of **Tetramer**


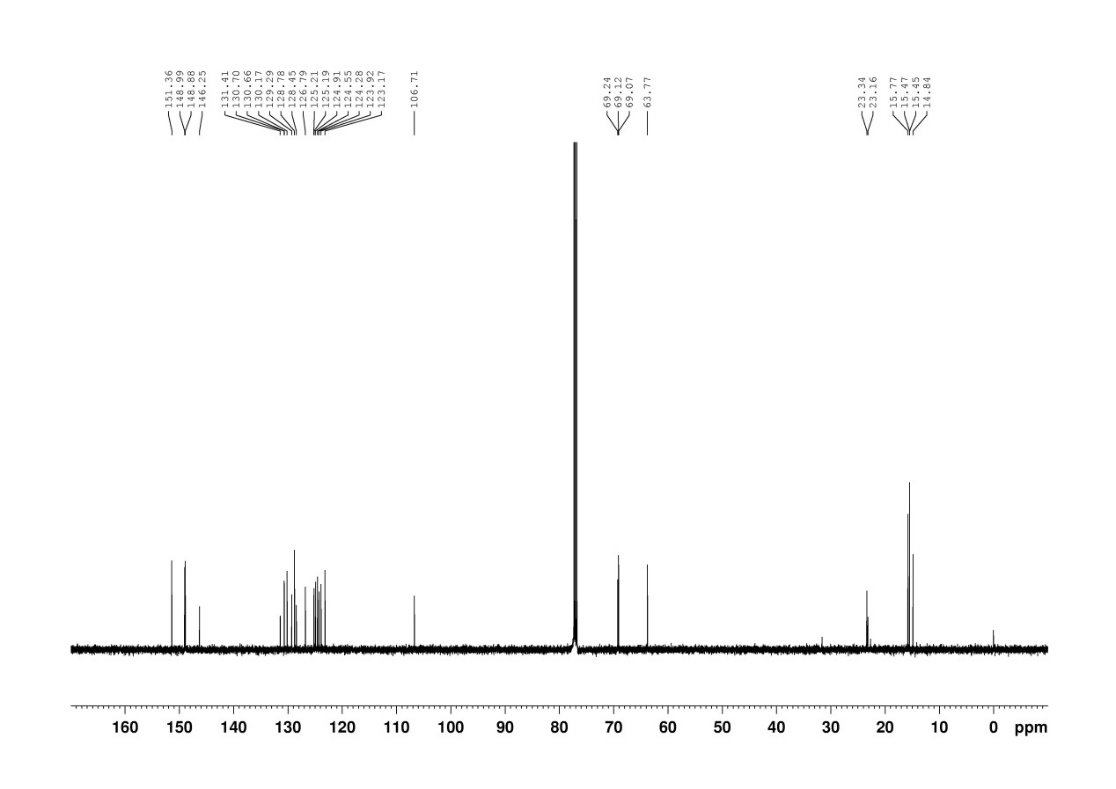


**Supplementary Figure 9.** ^13^C NMR spectrum (125 MHz, CDCl_3_, 298K) of **Tetramer**


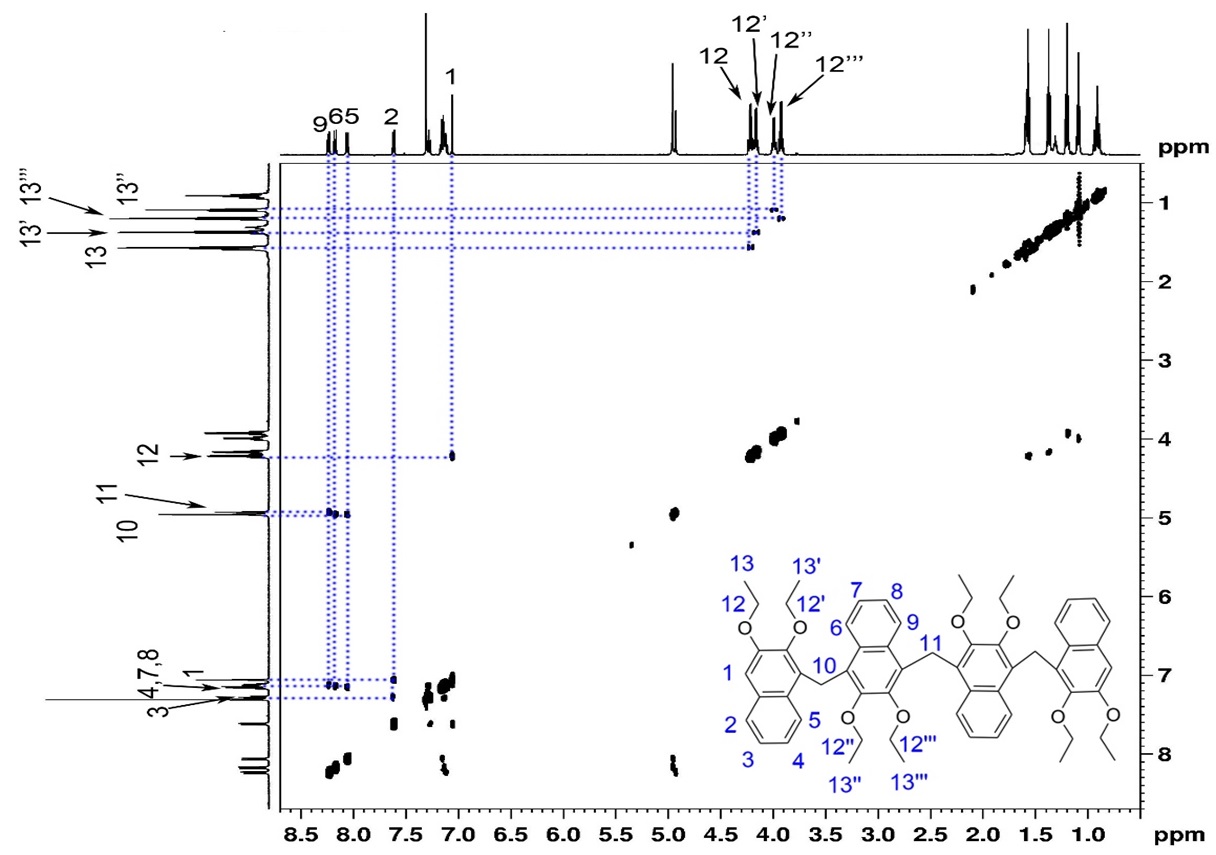


**Supplementary Figure 10.** 2D ^1^H,^1^H-NOESY spectrum (500 MHz, CDCl_3_, 298K) of **Tetramer**


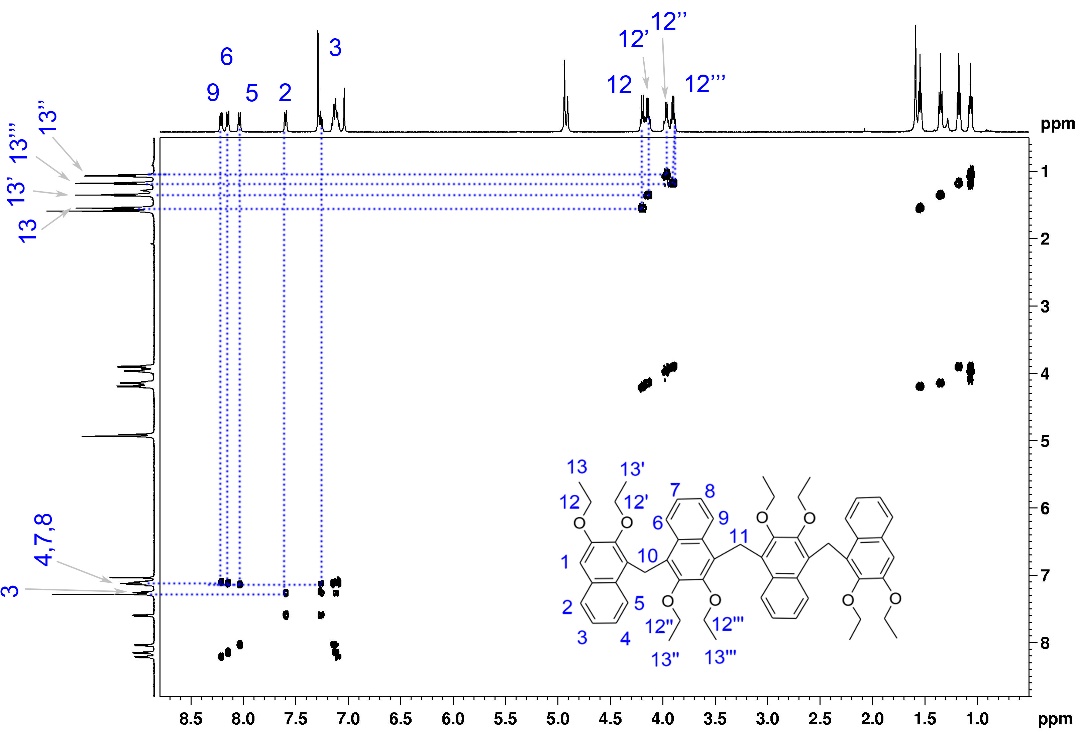


**Supplementary Figure 11.** 2D ^1^H,^1^H-COSY spectrum (500 MHz, CDCl_3_, 298K) of **Tetramer**

1. X-ray crystal data of oligomers **Dimer, Trimer and Tetramer.**

**Dimer** ：colorless，C_29_ H_32_O_4_，FW 443.54，Monoclinic，space group P21/n，a = 12.165(12) Å，b = 15.587(16) Å，c = 13.208(13) Å，α= 90°，β= 98.279(12) °，γ = 90°，V= 2478(4) Å^3^ ，Z = 4，*Dc*=1.189 Mg/m^3^ ，T=296(2) K，μ =0.078 mm^-1^; 12298 measured reflections, 4385 independent reflections, 303 parameters, 0 restraint, F(000)= 948，R_1_=0.0643, wR_2_ =0.1803 (all data), R_1_=0.1027，wR_2_ =0.2132 (all data)，max. residual density 0.458 e.Å^-3^; and goodness-of-fit (F^2^) =1.055. 151021a_0m. CCDC 1963983.

**Trimer**：colorless, C_44_ H_48_O_6_, FW 672.82, Monoclinic, space group C2/c*,* a = 29.365(2) Å, b = 10.1185(8) Å, c = 28.030(2) Å, α= 90°, β= 114.1350(10)°, γ = 90°, V = 7600.5(11) Å^3^, Z = 8, *Dc* = 1.176 Mg/m^3^, T = 296(2) K, μ =0.077 mm^-1^; 19245measured reflections, 6704 independent reflections, 458 parameters, 1 restraint, F(000) = 2880, R_1_=0.0632, wR_2_ =0.1753, R_1_= 0.1220, wR_2_ =0.2164 (all data)，max. residual density 0.435 e. Å^-3^; and goodness-of-fit (F^2^) =1.055. 150706b_0m. CCDC 1963984.

**Tetramer**：colorless，C_59_ H_68_O_10_，FW 937.13，Monoclinic，space group C2/c，a = 14.546(7) Å，b = 14.853(7) Å，c = 24.904(12) Å，α= 90 °，β= 98.161(6)°，γ = 90 °，V=5326(4) Å^3^ ，Z = 4，*Dc* = 1.169 Mg/m^3^ ，T = 296(2) K，μ =0.079mm^-1^; 13635 measured reflections, 4711 independent reflections, 317 parameters, 0 restraint, F(000)= 2008，R_1_=0.1038, wR_2_ =0.3396, R_1_=0.1267，wR_2_ =0.3695(all data)，max. residual density0.232 e. Å ^-3^; and goodness-of-fit (F^2^) =1.469. 150505b_0m. CCDC 1963985.


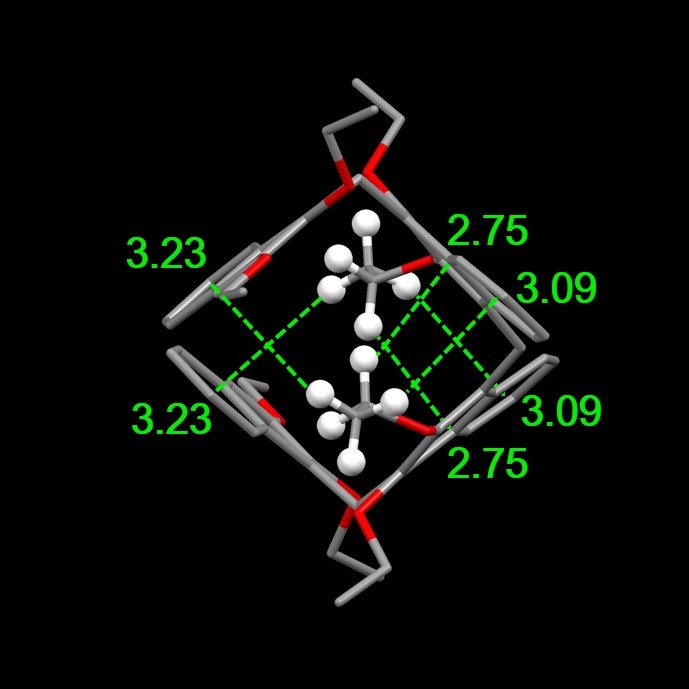

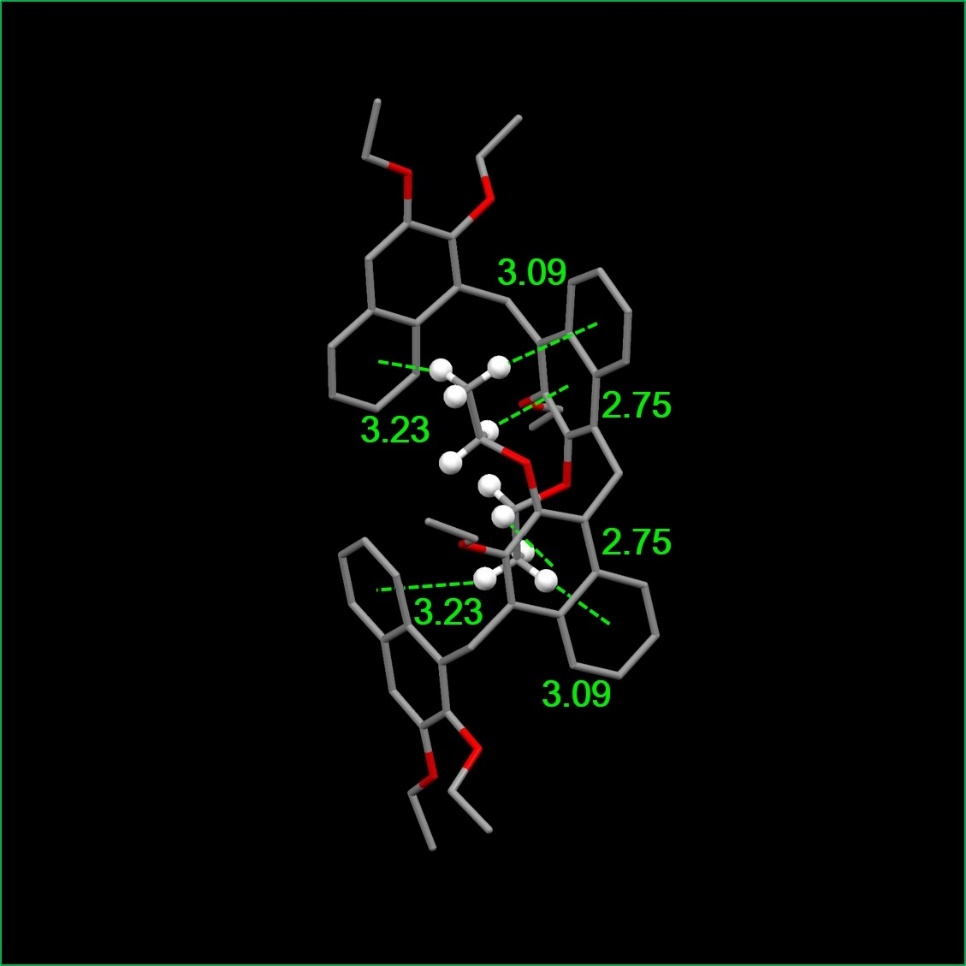


**Supplementary Figure 12.** Crystal structure of **Tetramer**. Left: top view; Right: side view. Oxygens are shown in red, carbons in gray, and hydrogens in white. Dashed lines represent C−H•••π interactions.

3. NOESY spectrum of **1**^+^•**Tetramer**


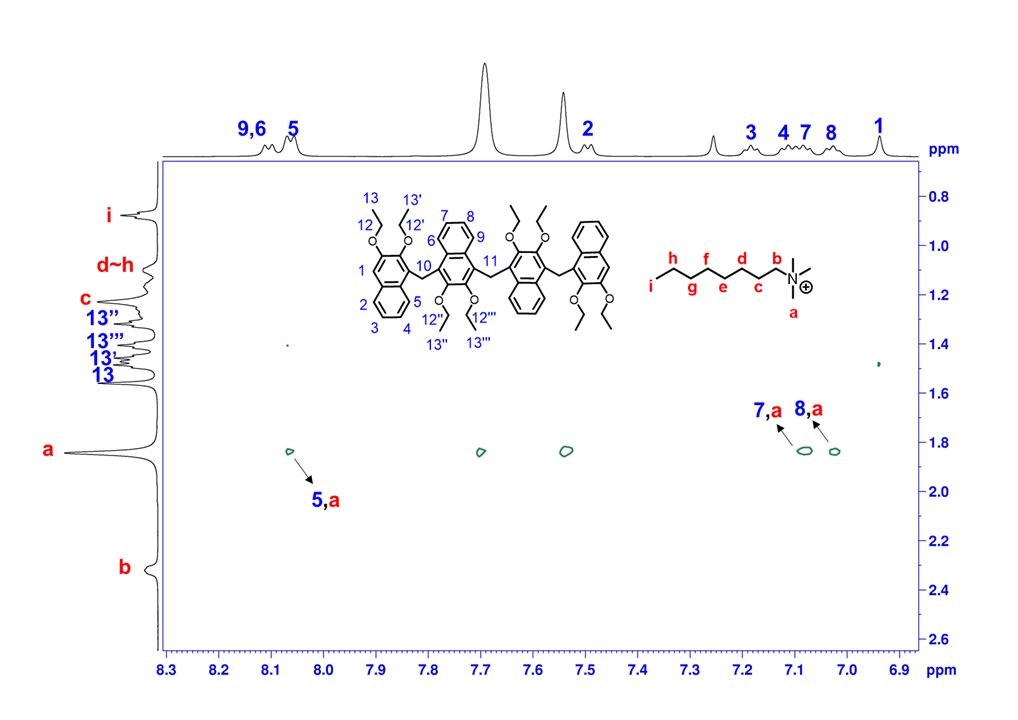


**Supplementary Figure 13**. 2D NOESY (600 MHz, CDCl_3_) of **1**•BArF (20 mM) and **Tetramer** (15 mM).

4. Mass spectrum of **1**^+^•**Tetramer**


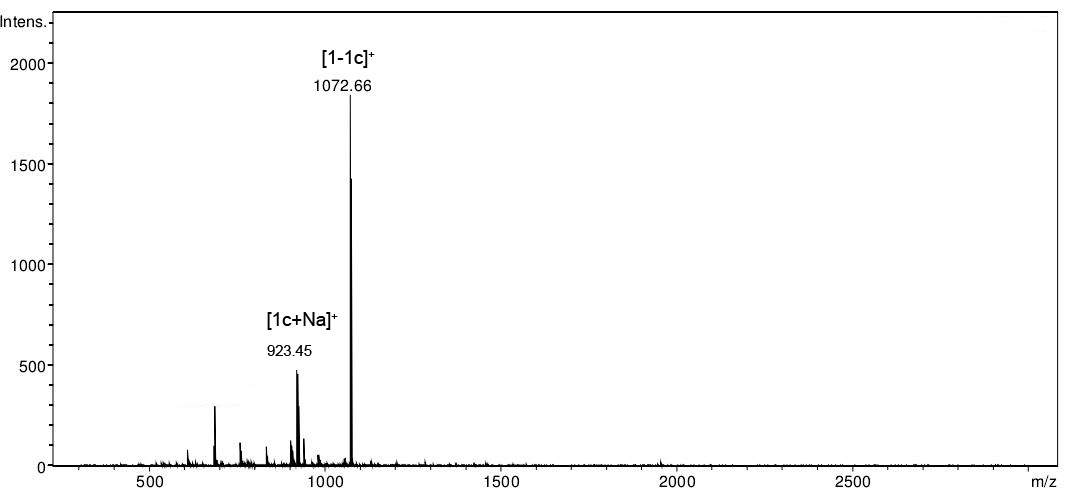


**Supplementary Figure 14**. ESI mass spectrum of an equimolar mixture of **1**•BArF and **Tetramer** in methanol solution. The concentration of host/guest is about 0.5 μmol L^−1^.

5. Additional ^1^H NMR spectra of host-guest mixture


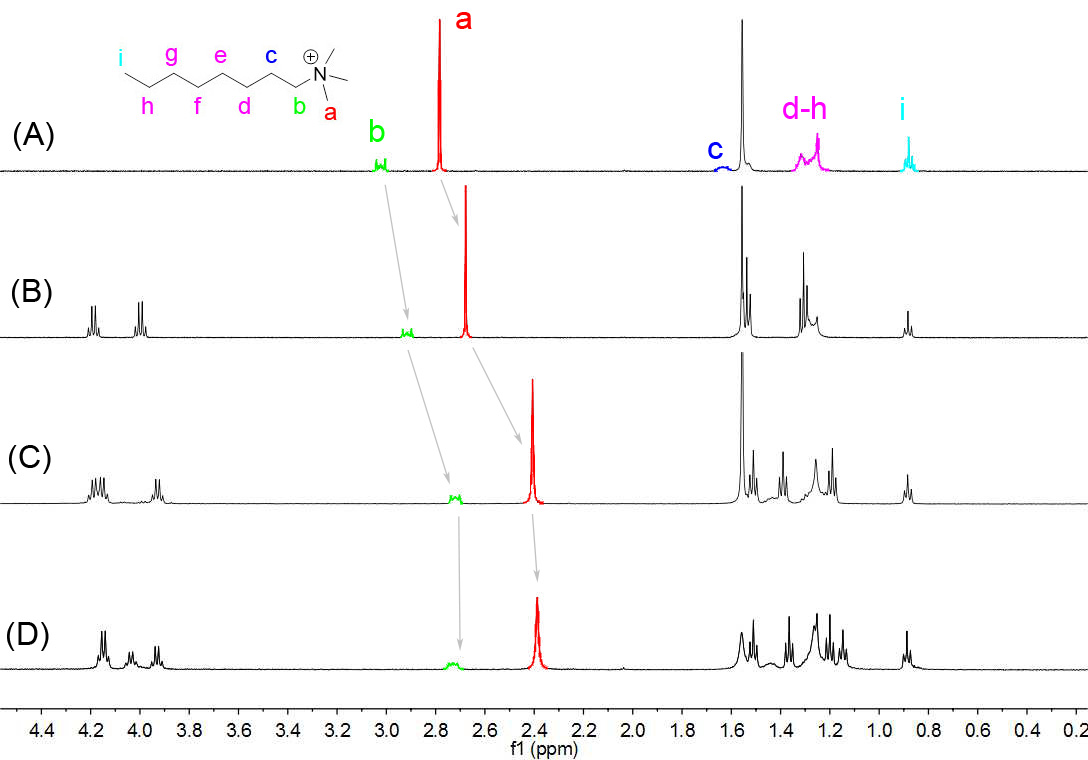


**Supplementary Figure 15**. ^1^H NMR spectra recorded (CDCl_3_, 298 K, 1.0–1.2 mmol) for: (A) **1^+^**; (B) **Dimer** + **1^+^**; (C) **Trimer** + **1^+^**; (D) **Tetramer** + **1^+^.**


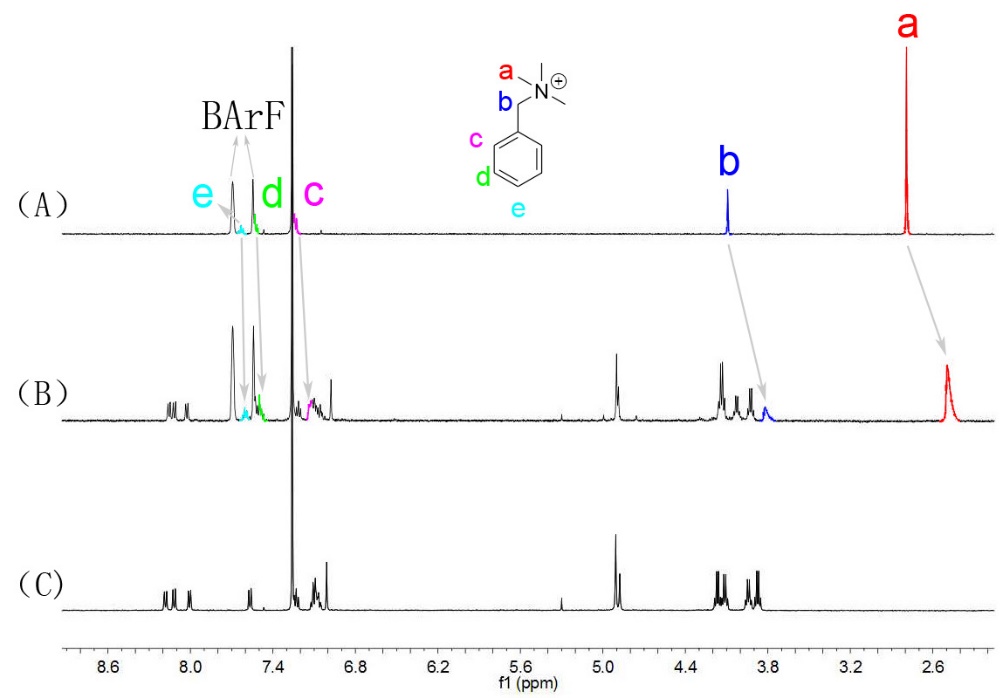


**Supplementary Figure 16**. ^1^H NMR spectra recorded (CDCl_3_, 298 K, 1.0–1.2 mmol) for: (A) **2^+^**; (B) **Tetramer** + **2^+^**; (C) **Tetramer.**


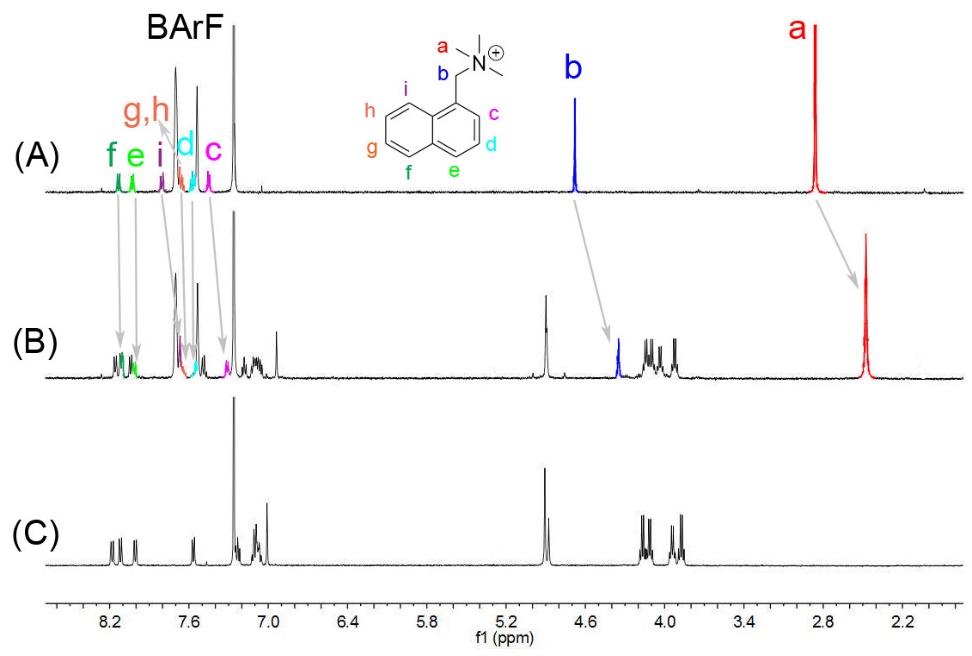


**Supplementary Figure 17**. ^1^H NMR spectra recorded (CDCl_3_, 298 K, 1.0–1.2 mmol) for: (A) **3^+^**; (B) **Tetramer** + **3^+^**; (C) **Tetramer.**


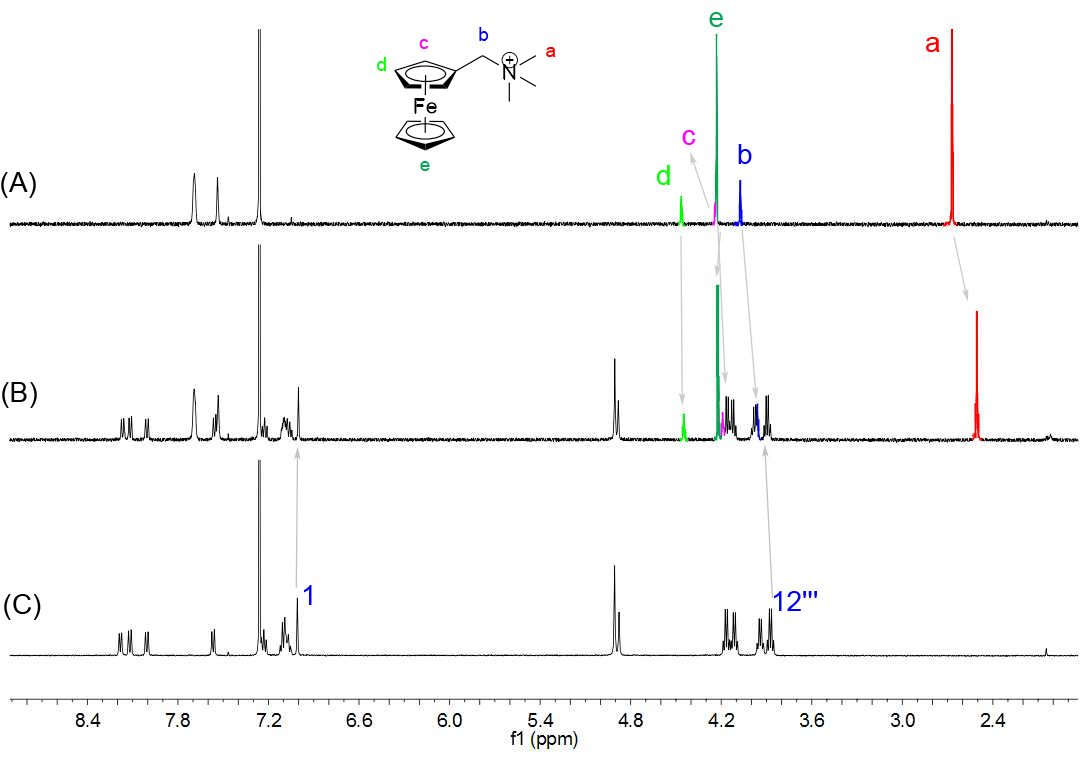


**Supplementary Figure 18**. ^1^H NMR spectra recorded (CDCl_3_, 298 K, 1.0–1.2 mmol) for: (A) **6^+^**; (B) **Tetramer** + **6^+^**; (C) **Tetramer.**


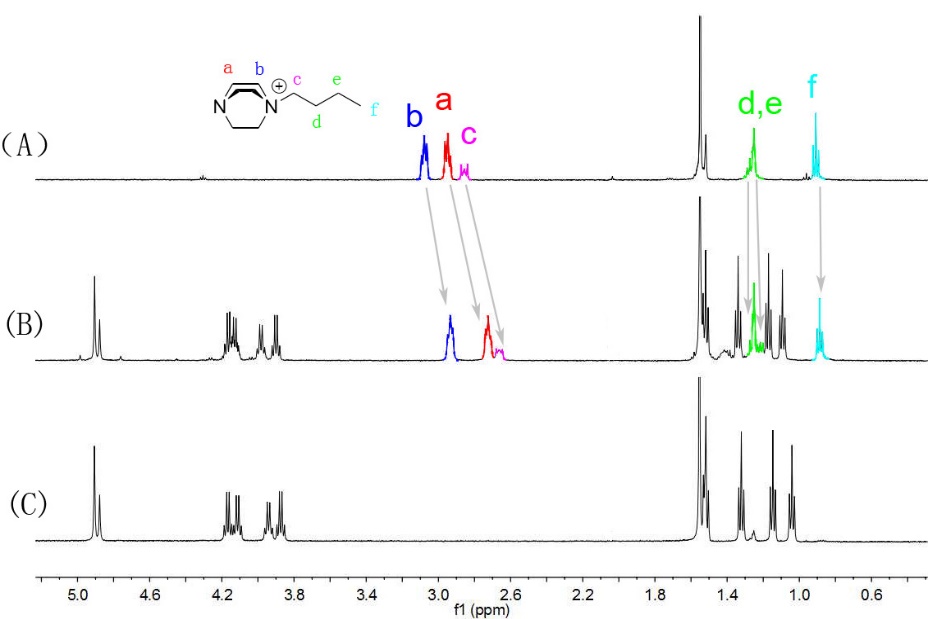


**Supplementary Figure 19**. ^1^H NMR spectra recorded (CDCl_3_, 298 K, 1.0–1.2 mmol) for: (A) **7^+^**; (B) **Tetramer** + **7^+^**; (C) **Tetramer.**


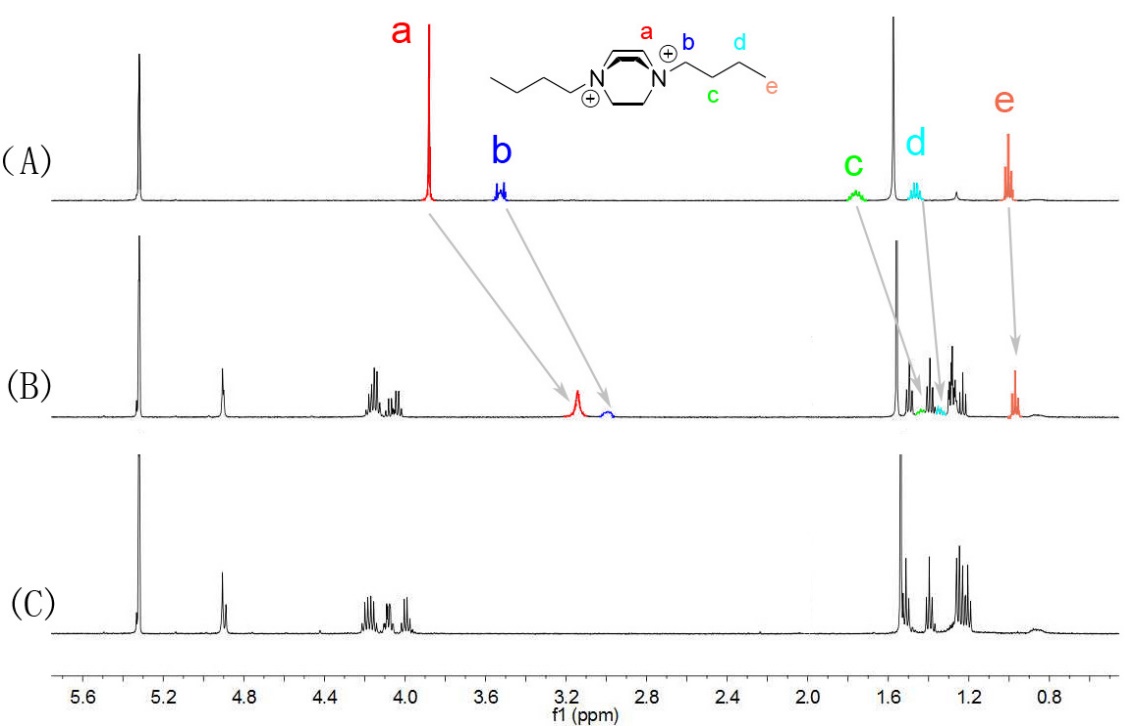


**Supplementary Figure 20**. ^1^H NMR spectra recorded (CD_2_Cl_2_, 298 K, 1.0–1.2 mmol) for: (A) **8^2+^**; (B) **Tetramer** + **8^2+^**; (C) **Tetramer.**


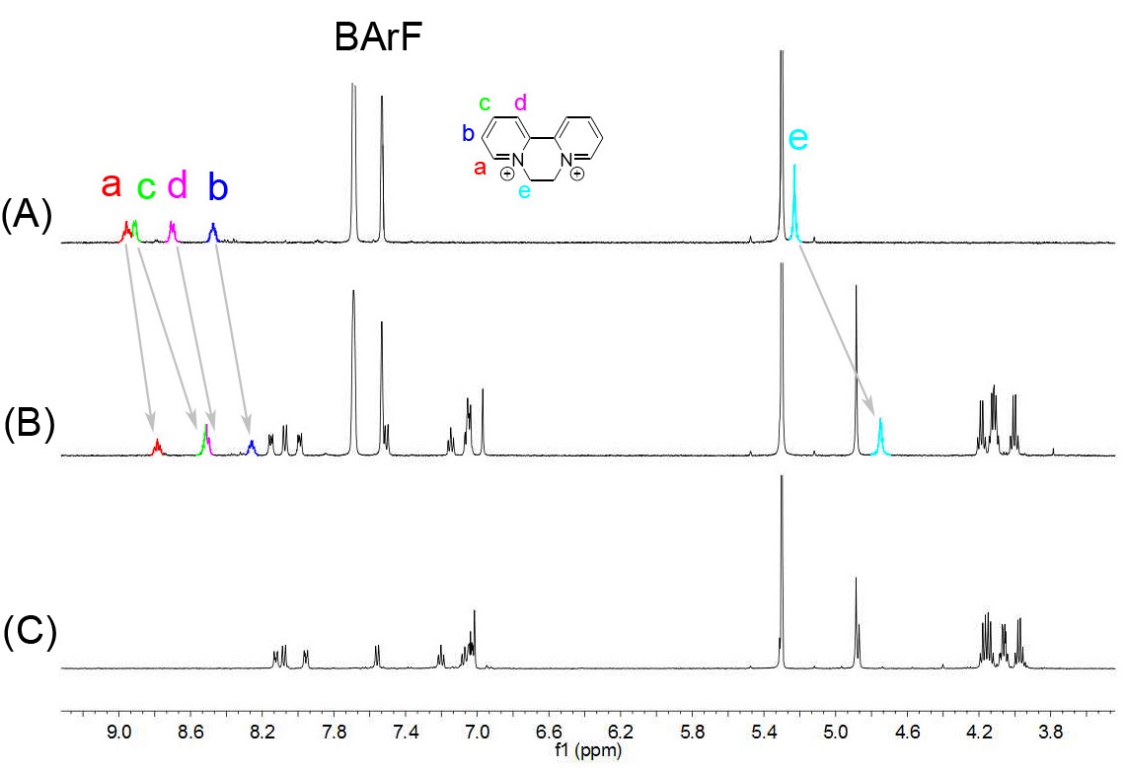


**Supplementary Figure 21**. ^1^H NMR spectra recorded (CD_2_Cl_2_, 298 K, 1.0–1.2 mmol) for: (A) **9^2+^**; (B) **Tetramer** + **9^2+^**; (C) **Tetramer.**


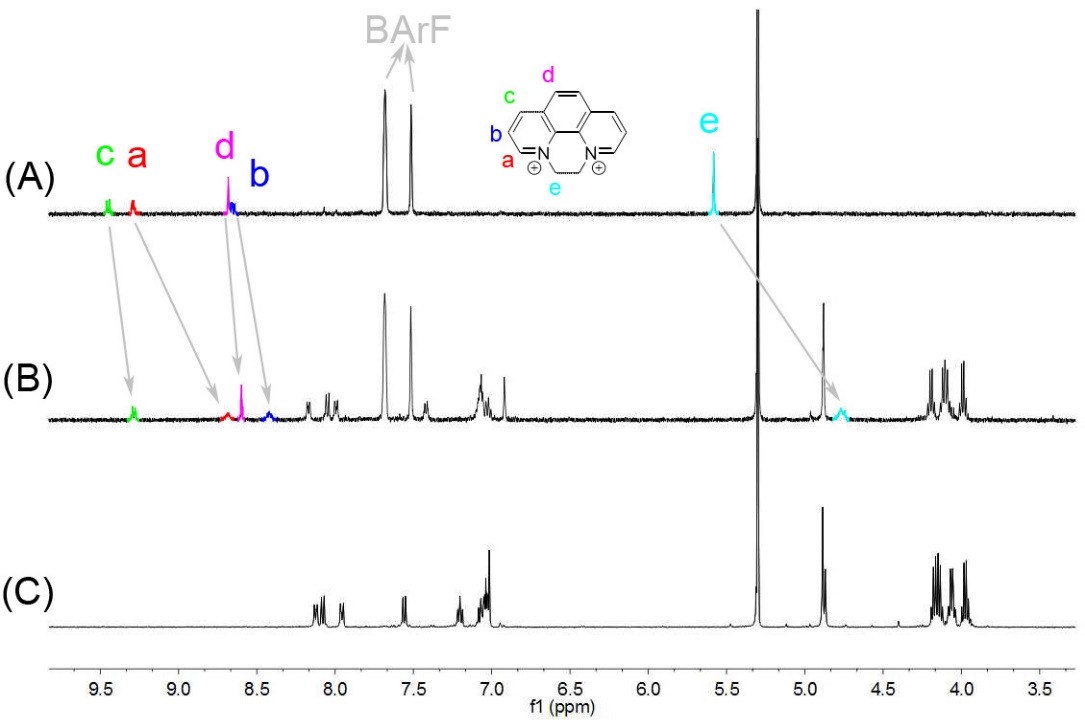


**Supplementary Figure 22**. ^1^H NMR spectra recorded (CD_2_Cl_2_, 298 K, 1.0–1.2 mmol) for: (A) **10^2+^**; (B) **Tetramer** + **10^2+^**; (C) **Tetramer.**


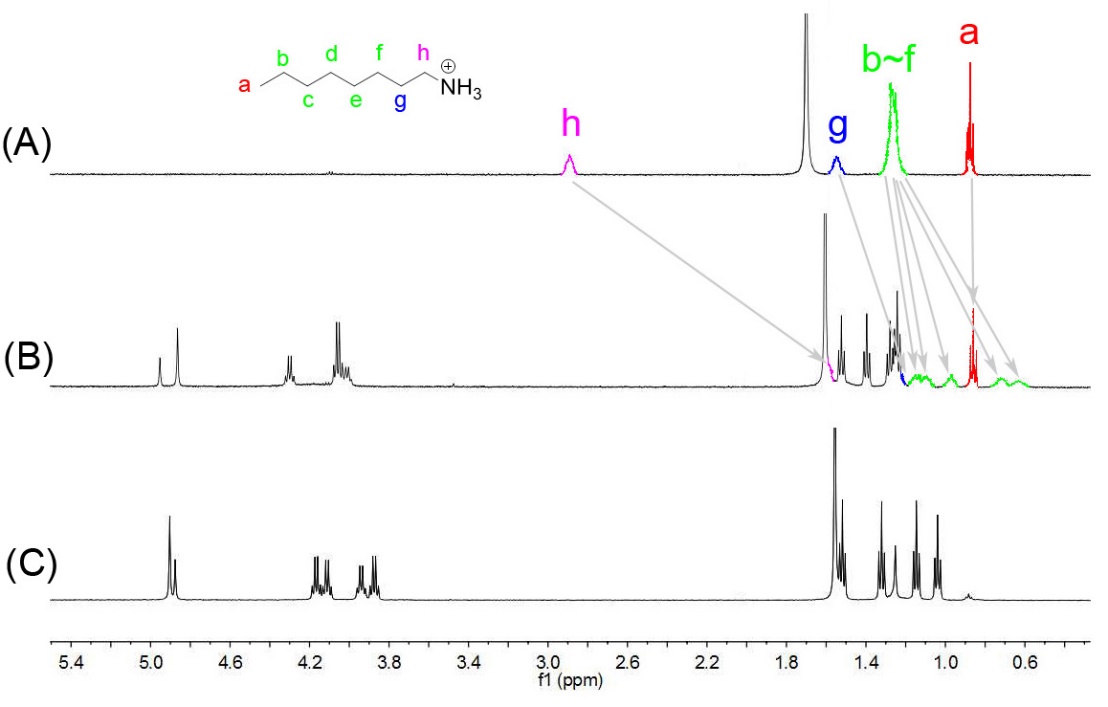


**Supplementary Figure 23**. ^1^H NMR spectra recorded (CDCl_3_, 298 K, 1.0–1.2 mmol) for: (A) **11^+^**; (B) **Tetramer** + **11^+^**; (C) **Tetramer.**


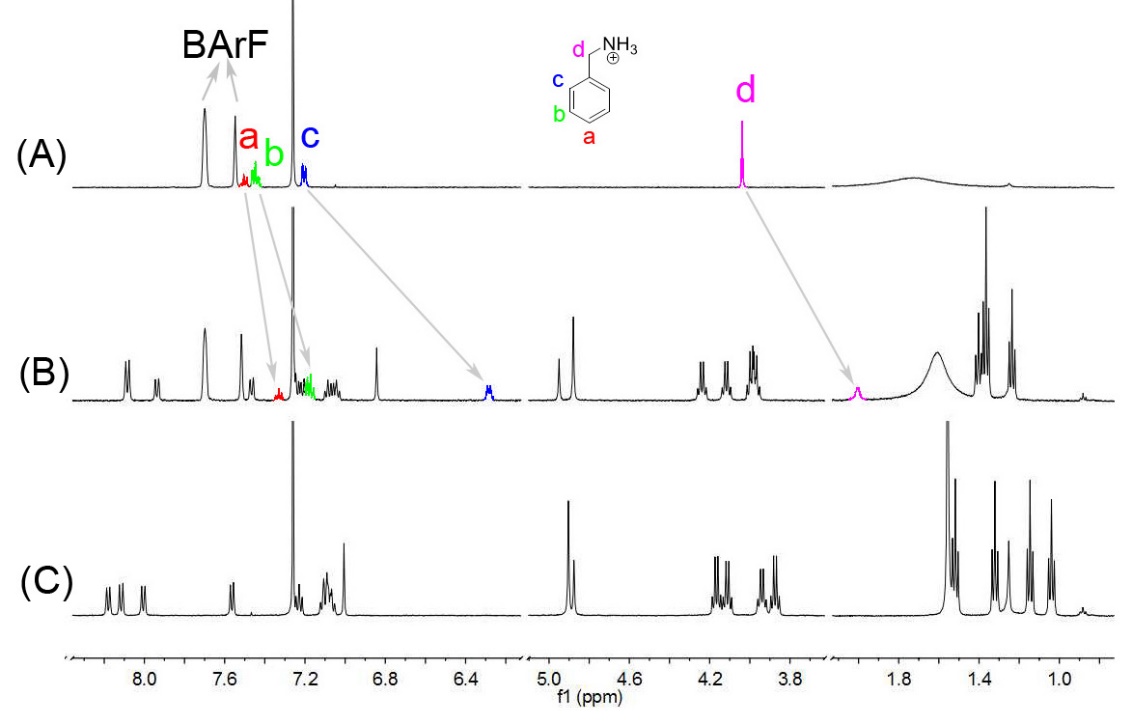


**Supplementary Figure 24**. ^1^H NMR spectra recorded (CDCl_3_, 298 K, 1.0–1.2 mmol) for: (A) **12^+^**; (B) **Tetramer** + **12^+^**; (C) **Tetramer.**


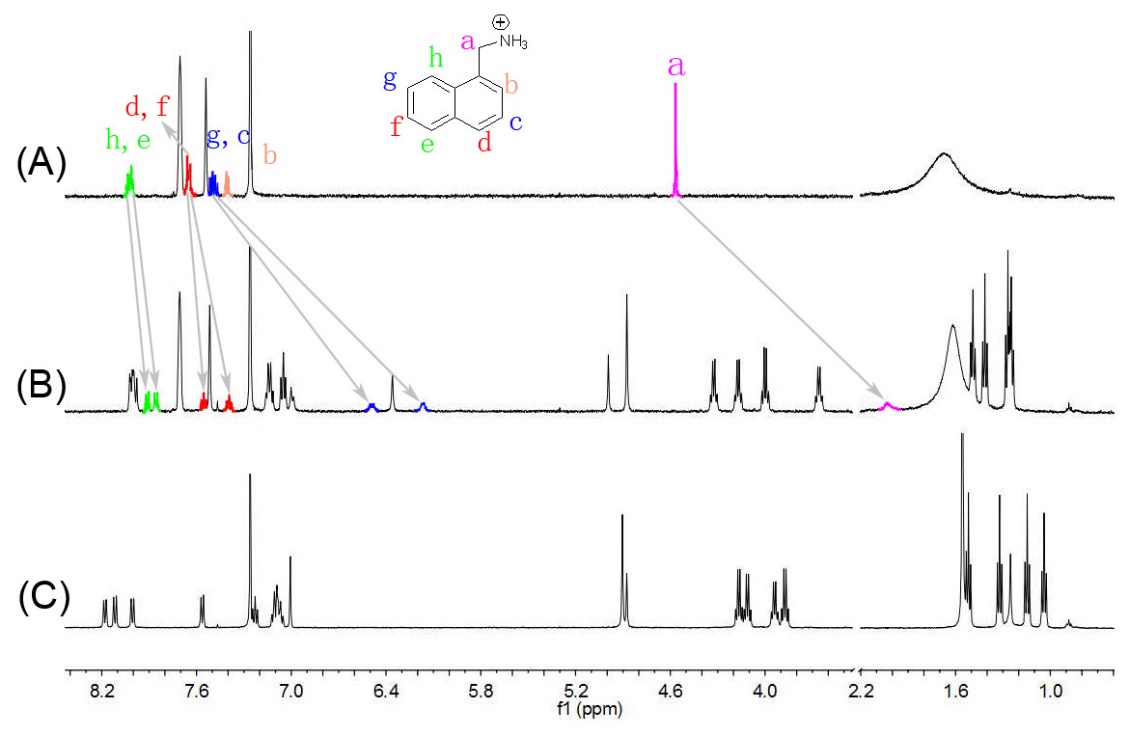


**Supplementary Figure 25**. ^1^H NMR spectra recorded (CDCl_3_, 298 K, 1.0–1.2 mmol) for: (A) **13^+^**; (B) **Tetramer** + **13^+^**; (C) **Tetramer.**


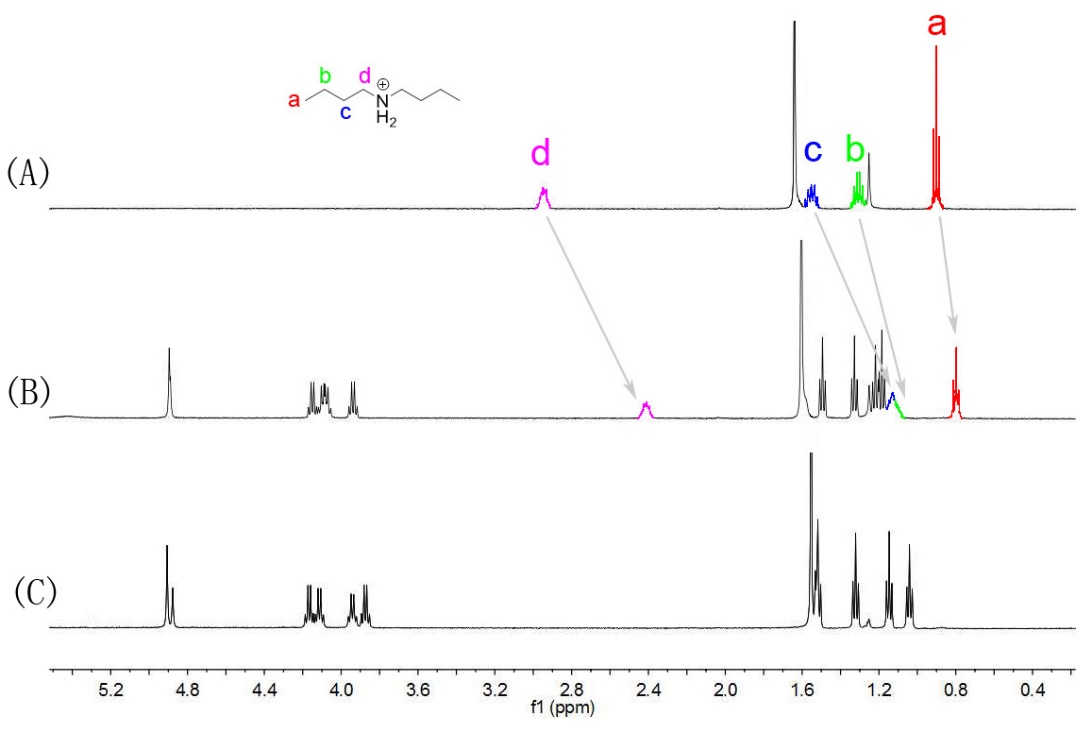


**Supplementary Figure 26**. ^1^H NMR spectra recorded (CDCl_3_, 298 K, 1.0–1.2 mmol) for: (A) **14^+^**; (B) **Tetramer** + **14^+^**; (C) **Tetramer.**


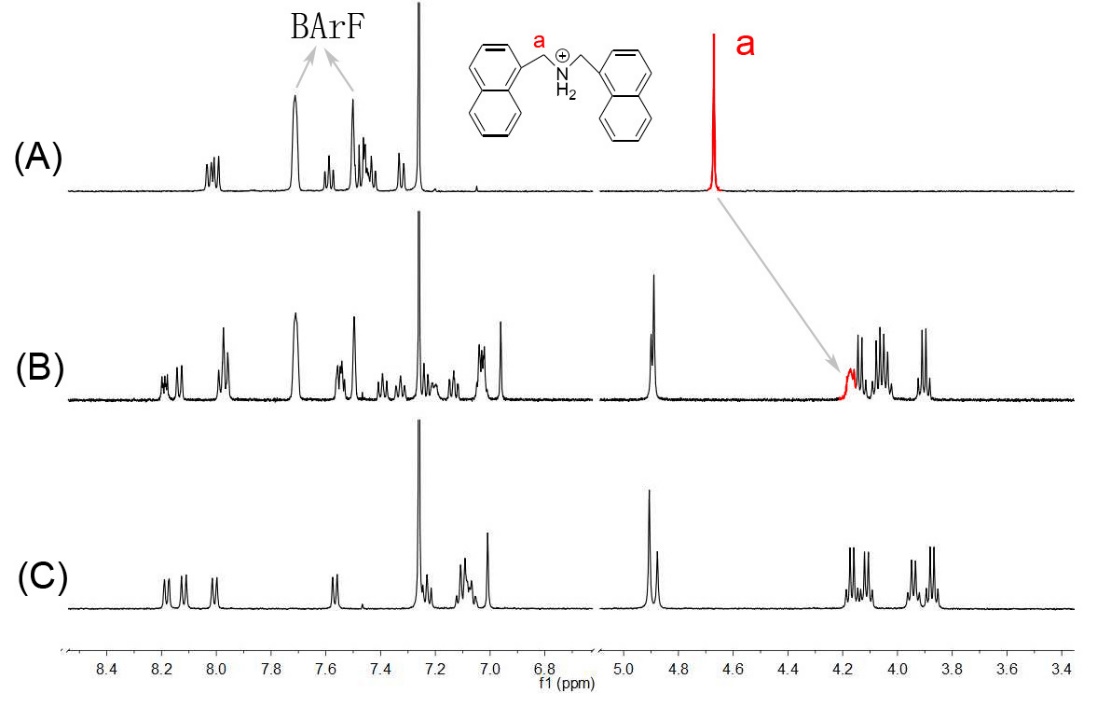


**Supplementary Figure 27**. ^1^H NMR spectra recorded (CDCl_3_, 298 K, 1.0–1.2 mmol) for: (A) **15^+^**; (B) **Tetramer** + **15^+^**; (C) **Tetramer.**


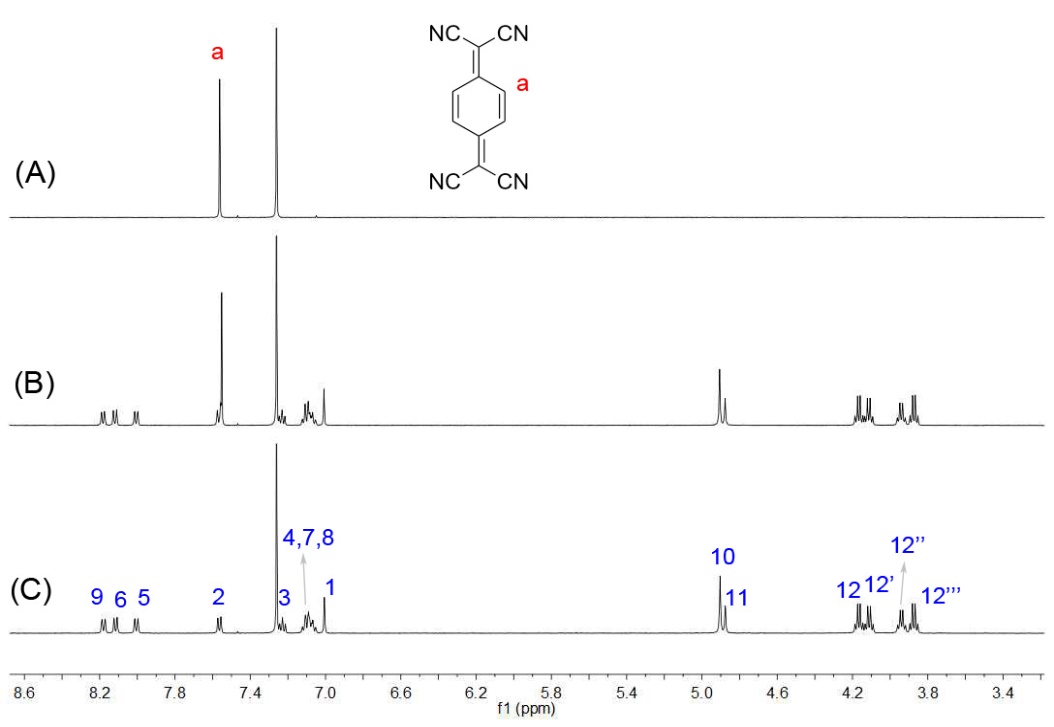


**Supplementary Figure 28**. ^1^H NMR spectra recorded (CDCl_3_, 298 K, 1.0–1.2 mmol) for: (A) **16**; (B) **Tetramer** + **16**; (C) **Tetramer.**


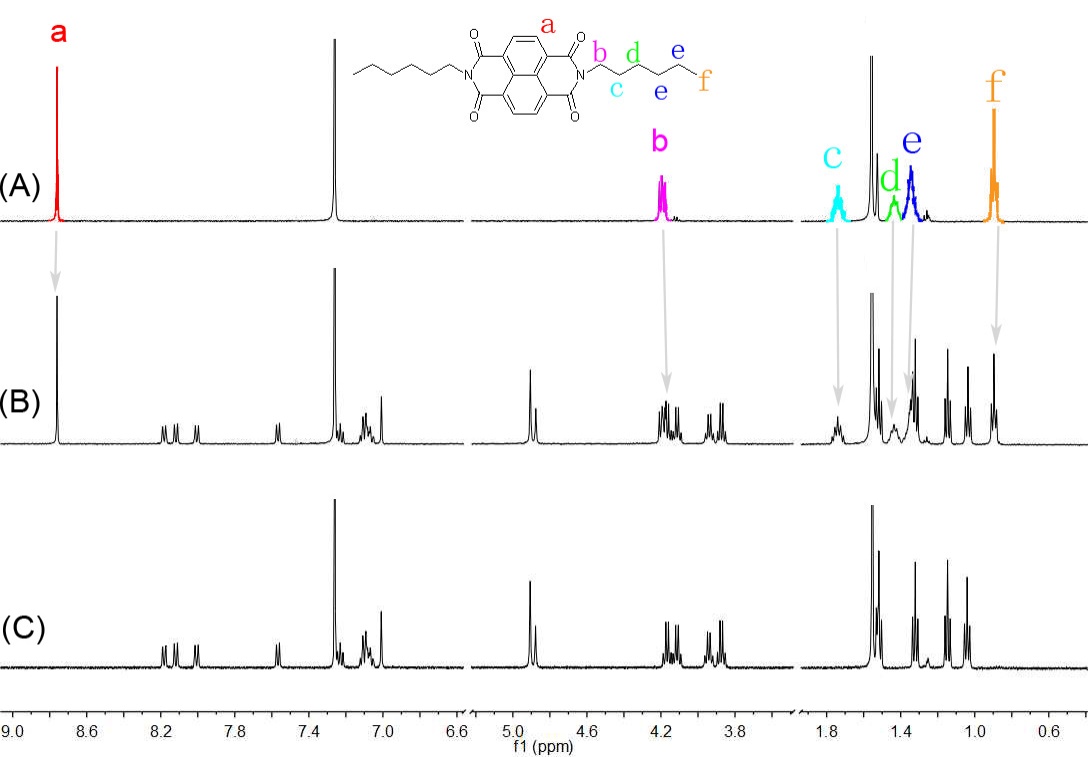


**Supplementary Figure 29**. ^1^H NMR spectra recorded (CDCl_3_, 298 K, 1.0–1.2 mmol) for: (A) **17**; (B) **Tetramer** + **17**; (C) **Tetramer.**

6. Determination of the association constants.

In the present host-guest systems, chemical exchange is fast on the NMR time scale. To determine the association constants (*K*_a_), ^1^H NMR titrations were performed in CDCl_3_ or CD_2_Cl_2_ with solutions which had a constant concentration of the host and varying concentrations of guest. The *K*_a_ values could be calculated by analyzing the sequential changes in chemical shift changes of the host that occurred with changes in guest concentration by using the nonlinear curve-fitting method from the following equation^[S1]^:

*A* = (*A*B_∞B_/[H]B_0B_) (0.5[G]B_0B_ + 0.5([H]B_0B_+1/*K*B_aB_)−(0.5 ([G]B_0PB_^2P^+(2[G]B_0B_(1/*K*B_aB_ − [H]B_0B_)) + (1/*K*B_aB_ + [H]B_0B_)P^2P^) P^0.5P^))

Where *A* is the chemical shift change of aromatic protons on the host at [G]B_0B_, *A*B_∞B_ is the chemical shift change when the host is completely complexed, [H]B_0B_ is the fixed initial concentration of the host, and [G]B_0B_ is the initial concentration of guest.


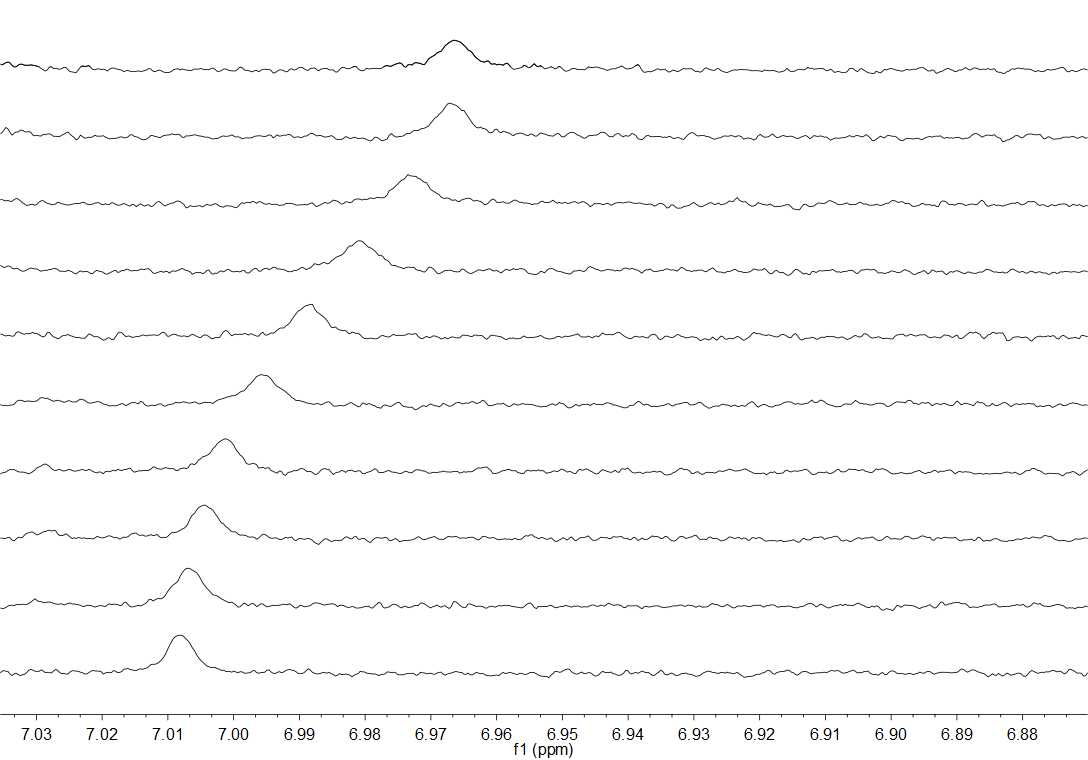

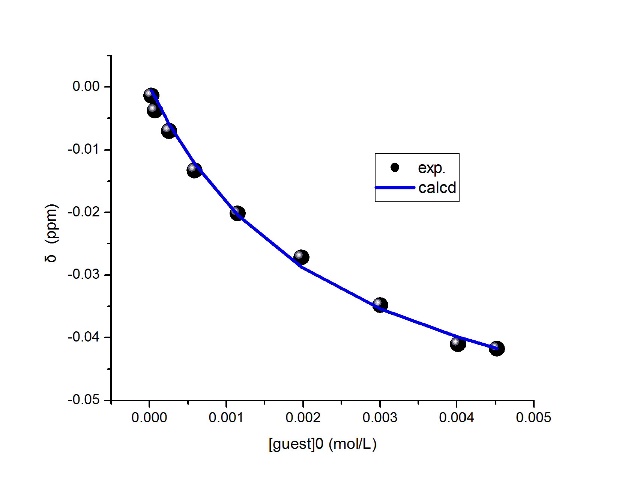


**Supplementary Figure 30**. The non-linear curve-fitting (NMR titrations) for the complexation of **Tetramer** (0.50 × 10^−3^ mol/L) with and guest **1**^+^ in CDCl_3_ at 298 K.

7. Optimized structures, cartesian coordinates and computed total energies of **1^+^**•**Tetramer** by Gaussian 03 program. ^[S2]^

PM6 in gas phase, E = -571.2 kJ/mol, Charge = 1, Multiplicity = 1


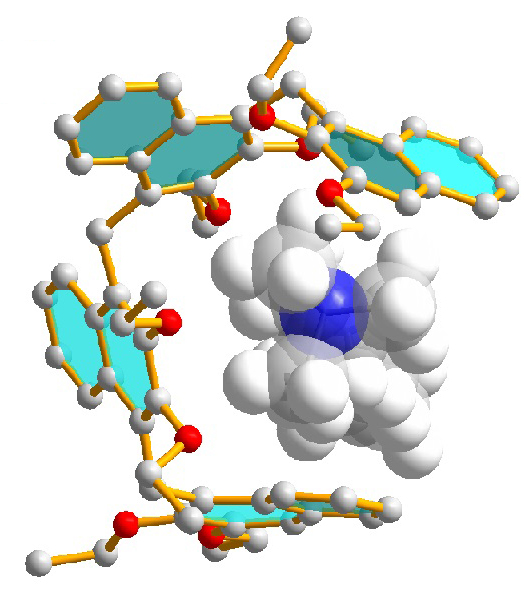


C -5.42857100 1.23992000 -0.84310600

C -4.93148500 2.56805300 -0.56028900

C -5.21164700 3.17771400 0.69172300

C -5.96587100 2.47273400 1.68114000

C -6.46962100 1.22964800 1.39248400

C -6.22456800 0.61416800 0.09955400

C -4.20201700 3.32603800 -1.52535200

C -3.76866100 4.60282300 -1.25670200

C -4.04558900 5.20156900 0.00162800

C -4.74617000 4.50307100 0.95560200

C -4.00574300 -0.43253000 -2.11252500

C -4.25502100 -1.83810700 -1.90318500

C -3.17346300 -2.74306000 -1.72699900

C -1.80875400 -2.27754200 -1.82138400

C -1.59558700 -0.96045400 -2.17391600

C -2.69486600 -0.03491300 -2.29572200

C -5.58789300 -2.35871900 -1.91067900

C -5.83967500 -3.69259800 -1.71082000

C -4.76056000 -4.59096000 -1.49017100

C -3.46975000 -4.12530800 -1.50009600

C 2.68134300 -2.18736400 1.01190100

C 2.91297300 -2.66533400 -0.33203300

C 1.81856200 -2.99373100 -1.18153800

C 0.45515100 -2.83282100 -0.72845300

C 0.25793600 -2.38954100 0.56250500

C 1.36717000 -2.08699300 1.42780500

C 4.23632800 -2.87666300 -0.82969500

C 4.46991500 -3.37043300 -2.08864100

C 3.37799200 -3.70148900 -2.93389600

C 2.09461200 -3.52070300 -2.48437000

C -0.67984900 -3.25171400 -1.62496900

C -5.13748900 0.56514800 -2.16308900

C 4.60580900 -0.66538400 1.78328200

C 4.11395000 0.62354400 2.20820700

C 4.89533400 1.78740100 1.97552200

C 6.15657200 1.68441200 1.30706900

C 6.65511000 0.44983700 0.98293400

C 5.89832400 -0.74945000 1.28530000

C 2.90271200 0.76997200 2.94696800

C 2.50129800 1.99586000 3.42579900

C 3.27160600 3.15926600 3.16192100

C 4.43950800 3.05745800 2.44479500

C 3.80507100 -1.92802800 1.98686100

O -1.00964300 -2.13960900 1.08261200

C -3.12091100 -2.87709900 1.84598200

C -1.70912600 -3.34092900 1.56559600

O 1.06283900 -1.61753400 2.70597000

C 0.83815400 -1.93135400 5.02488300

C 0.89923600 -2.67940300 3.71085800

O 7.85553700 0.21449500 0.35980200

C 9.85911600 0.74387200 -0.71710600

C 8.64778700 1.37660000 -0.06727000

O 6.36220700 -2.03173600 1.10165700

C 7.92148500 -3.75639800 0.93283500

C 7.78558000 -2.25353900 0.80633000

O -7.19509400 0.44810600 2.24884100

C -8.51498800 -0.04802700 4.10967900

C -7.64895800 1.04245100 3.51630400

O -6.69502400 -0.64550900 -0.20314900

C -9.00335000 -0.07020100 -0.81177900

C -8.10780500 -0.92649800 0.05762200

O -0.32237900 -0.42369200 -2.32114800

C 1.72865200 -0.24185500 -3.46741200

C 0.32152400 -0.77538100 -3.60037200

O -2.42949200 1.31974800 -2.44238200

C -0.96142800 2.88272500 -3.43716300

C -1.89436100 1.73172100 -3.74887200

H -6.14461100 2.95880600 2.63542100

H -3.99482600 2.86301400 -2.49637700

H -3.23093300 5.17914100 -2.00779600

H -3.70887400 6.22118600 0.18866900

H -4.98349100 4.95953600 1.91558000

H -6.41410200 -1.65617400 -2.07805300

H -6.85762500 -4.08012300 -1.72412200

H -4.97536300 -5.64674100 -1.32521600

H -2.63828900 -4.81896100 -1.34689500

H 5.08982800 -2.65079300 -0.17828100

H 5.48691000 -3.52248700 -2.44908100

H 3.57558200 -4.10460300 -3.92685800

H 1.25107900 -3.78951900 -3.12782300

H -1.07015800 -4.22287400 -1.22327700

H -0.29433500 -3.51677400 -2.64469700

H -6.07216700 0.05783300 -2.51066000

H -4.90893000 1.33292700 -2.93955300

H 6.69835600 2.60103000 1.08908900

H 2.30283300 -0.12523400 3.15042800

H 1.60367100 2.08843800 4.03304900

H 2.93455900 4.11966400 3.54744100

H 5.04794600 3.93829200 2.24302100

H 3.39770700 -1.91300000 3.02644700

H 4.49814500 -2.80787500 1.96874300

H -3.62759300 -2.53605200 0.92841100

H -3.72951300 -3.69230000 2.26045500

H -3.14612800 -2.04813400 2.56277600

H -1.66042800 -4.12175400 0.78909300

H -1.17280300 -3.67056500 2.48160100

H 0.69049700 -2.62061100 5.86728400

H 1.76531100 -1.37223100 5.21782200

H 0.01733300 -1.20378300 5.04103800

H -0.04246600 -3.21694200 3.47019100

H 1.75723800 -3.36918100 3.64535000

H 10.54888600 1.50897400 -1.09740900

H 10.41788400 0.11625400 -0.00872900

H 9.57720500 0.09943700 -1.56109100

H 8.02948800 1.96769900 -0.78265300

H 8.89801100 1.97418100 0.82518500

H 8.95043800 -4.07798900 0.72754800

H 7.65986600 -4.10110200 1.94307000

H 7.25853500 -4.28560000 0.23559300

H 7.97685600 -1.87827300 -0.21523200

H 8.38977500 -1.69595800 1.54226100

H -8.82352500 0.20189700 5.13353300

H -9.42707900 -0.21319500 3.51973900

H -7.98254300 -1.00954800 4.14692400

H -6.75660700 1.26334400 4.12547700

H -8.20998500 1.96547300 3.29033900

H -10.05601000 -0.36653400 -0.71463700

H -8.93940300 0.99265800 -0.54199700

H -8.73807800 -0.14679600 -1.87368600

H -8.13342100 -1.99935700 -0.21358600

H -8.31014500 -0.80507100 1.13856500

H 2.19916000 -0.08717300 -4.44486100

H 1.73527800 0.72749500 -2.92938600

H 2.37339100 -0.91905800 -2.88658800

H 0.28015000 -1.86736900 -3.73892500

H -0.26132900 -0.26407800 -4.39540200

H -0.62529100 3.38412800 -4.35372700

H -1.45069400 3.63617100 -2.80507500

H -0.06791000 2.54018900 -2.89535900

H -1.37841200 0.88502200 -4.24200700

H -2.77590900 2.03243600 -4.34122800

C 7.47774900 4.51874000 -2.64975000

H 7.03322000 5.26068900 -3.32458600

H 7.73280600 5.04037300 -1.72064400

H 8.41540300 4.18712700 -3.11039600

C 6.53321900 3.34562600 -2.40470200

H 6.32276300 2.82866300 -3.36136000

H 7.03650500 2.58868100 -1.75691100

C 5.21966500 3.79789500 -1.75293300

H 5.43164400 4.27849500 -0.77843100

H 4.74440400 4.58117900 -2.37480200

C 4.25151300 2.62331400 -1.55916800

H 4.74806600 1.81901900 -0.97810900

H 3.99969600 2.17690700 -2.53941800

C 2.97373800 3.06760300 -0.83412200

H 2.48185500 3.88130400 -1.39960600

H 3.24122000 3.49907500 0.15230700

C 2.00558200 1.89454100 -0.64093000

H 2.53184200 1.04709200 -0.15152100

H 1.67567800 1.50940100 -1.63471300

C 0.79197400 2.30236000 0.20654800

H 0.43907300 3.30638300 -0.10177700

H 1.12118600 2.39865200 1.26343500

C -0.33592300 1.27917200 0.06038200

H 0.07252400 0.23690700 -0.01302000

H -0.87805200 1.43540900 -0.91494100

C -2.53847700 0.46471400 0.84614000

H -2.21797000 -0.57247600 0.58121800

H -3.26447500 0.38754500 1.66967900

H -3.07362300 0.87846400 -0.03270700

C -1.83495900 2.73562400 1.44234600

H -1.03931000 3.38314800 1.84398600

H -2.19861600 3.18977600 0.49625500

H -2.68355000 2.76313300 2.14899600

C -0.73213900 0.77613500 2.47231200

H -1.40719200 0.86270500 3.33422400

H -0.47794000 -0.31454700 2.35458900

H 0.21995100 1.28330900 2.71254200

N -1.34907000 1.32210900 1.20836600

**8. Reference**

[S1] a) K. A. Connors, Binding Constants; Wiley: New York, **1987**. Corbin, P. S. Ph.D. Dissertation, University of Illinois at Urbana-Champaign, Urbana, IL, 1999; b) R. P. Ashton, R. Ballardini, V. Balzani, M. Belohradsky, M. T. Gandolfi, D. Philp, L. Prodi, F. M. Raymo, M. V. Reddington, N. Spencer, J. F. Stoddart, M. Venturi , D. J. Williams, *J. Am. Chem. Soc.*, **1996**, *118*, 4931–4951; c) Y. Inoue, K. Yamamoto, T. Wada, S. Everitt, X.-M. Gao, Z.-J. Hou, L.-H. Tong, S.-K. Jiang, H.-M. Wu, *J. Chem. Soc., Perkin Trans. 2*, **1998**, 1807−1816.

[S2] M. J. Frisch, G. W. Trucks, H. B. Schlegel, G. E. Scuseria, M. A. Robb, J. R. Cheeseman, J. A., Jr. Montgomery, T. Vreven, K. N. Kudin, J. C. Burant, J. M. Millam, S. S. Iyengar, J. Tomasi, V. Barone, B. Mennucci, M. Cossi, G. Scalmani, N. Rega, G. A. Petersson, H. Nakatsuji, M. Hada, M. Ehara, K. Toyota, R. Fukuda, J. Hasegawa, M. Ishida, T. Nakajima, Y. Honda, O. Kitao, H. Nakai, M. Klene, X. Li, J. E. Knox, H. P. Hratchian, J. B. Cross, V. Bakken, C. Adamo, J. Jaramillo, R. Gomperts, R. E. Stratmann, O. Yazyev, A. J. Austin, R. Cammi, C. Pomelli, J. W. Ochterski, P. Y. Ayala, K. Morokuma, G. A. Voth, P. Salvador, J. J. Dannenberg, V. G. Zakrzewski, S. Dapprich, A. D. Daniels, M. C. Strain, O. Farkas, D. K. Malick, A. D. Rabuck, K. Raghavachari, J. B. Foresman, J. V. Ortiz, Q. Cui, A. G. Baboul, S. Clifford, J. Cioslowski, B. B. Stefanov, G. Liu, A. Liashenko, P. Piskorz, I. Komaromi, R. L. Martin, D. J. Fox, T. Keith, M. A. Al-Laham, C. Y. Peng, A. Nanayakkara, M. Challacombe, P. M. W. Gill, B. Johnson, W. Chen, M. W. Wong, C. Gonzalez, J. A. Pople, Gaussian 03, revision E.01, Gaussian, Inc., Wallingford, CT, **2004**.
